# DNA barcoding of coastal ray-finned fishes in Vietnam

**DOI:** 10.1371/journal.pone.0222631

**Published:** 2019-09-19

**Authors:** Pham The Thu, Wen-Chien Huang, Tak-Kei Chou, Nguyen Van Quan, Pham Van Chien, Fan Li, Kwang-Tsao Shao, Te-Yu Liao

**Affiliations:** 1 Institute of Marine Environment and Resources, Vietnam Academy of Science and Technology, Hai Phong, Vietnam; 2 Doctoral Degree Program in Marine Biotechnology, National Sun Yat-sen University, Kaohsiung, Taiwan; 3 Doctoral Degree Program in Marine Biotechnology, Academia Sinica, Taipei, Taiwan; 4 Department of Oceanography, National Sun Yat-sen University, Kaohsiung, Taiwan; 5 Biodiversity Research Center, Academia Sinica, Taipei, Taiwan; 6 Institute of Marine Biology, National Taiwan Ocean University, Keelung, Taiwan; National Cheng Kung University, TAIWAN

## Abstract

DNA barcoding based on a fragment of the cytochrome c oxidase subunit I (*COI*) gene is widely applied in species identification and biodiversity studies. The aim of this study was to establish a comprehensive barcoding database of coastal ray-finned fishes in Vietnam. A total of 3,638 specimens were collected from fish landing sites in northern, central and southern Vietnam. Seven hundred and sixty-five *COI* sequences of ray-finned fishes were generated, belonging to 458 species, 273 genera, 113 families and 43 orders. A total of 59 species were newly recorded in Vietnam and sequences of six species were new to the Genbank and BOLD online databases. Only 32 species cannot be annotated to species level because difficulty in morphological identifications and their Kimura-2-Parameter (K2P) genetic distances to most similar sequences were more than 2%. Moreover, intra-specific genetic distances in some species are also higher than 2%, implying the existence of putative cryptic species. The mean K2P genetic distances within species, genera, families, orders and classes were 0.34%, 12.14%, 17.39%, 21.42%, and 24.80, respectively. Species compositions are quite different with only 16 common species among northern, central and southern Vietnam. This may attribute to multiple habitats and environmental factors across the 3,260 km Vietnamese coastline. Our results confirmed that DNA barcoding is an efficient and reliable tool for coastal fish identification in Vietnam, and also established a reliable DNA barcode reference library for these fishes. DNA barcodes will contribute to future efforts to achieve better monitoring, conservation, and management of fisheries in Vietnam.

## Introduction

The DNA-based barcoding method has been proven to be a valuable molecular tool for species identification and it is accessible to non-specialists [1–3]. Currently, DNA barcoding has been employed to a large variety of organisms ranging from yeasts to humans with different standard region of genes for various organisms, including the gene of *COI* (cytochrome c oxidase 1) for animals [4] and it has been shown effective in discriminating fish species [5–7]. FISH-BOL [8] is also one of fish barcode database that is now well established and aims at DNA barcoding all the fishes of the world [8], with a total of approximately 7,800 fish species barcoded, among which 2019 species are distributed in Southeast Asia [9, 10]. When the reference sequence library is in place, new specimens and products can be identified by comparing their DNA barcode sequences against this barcode reference library. Hence, DNA barcoding has now been broadly applied to various fields such as preserving natural resources, protecting endangered species, controlling agriculture pests, identifying disease vectors, monitoring water quality, authentication of natural health products, identification of medicinal plants, food traceability and commercial-fraud prevention [4, 11].

Despite DNA barcoding has been successfully identified marine ichthyofauna and provided the wealth of DNA barcode information in many places, such as Portugal [12], Canada [6, 13], Australia [5], China [14–17], India [18], and Taiwan [19]. However, in Southeast Asian waters, an area exceptionally rich in fish biodiversity with more than 3,400 marine and 1000 freshwater species [20, 21], DNA barcoding studies have just been concentrated recently, such as 18 species in Laguna de Bay [22] and 27 commercially important species of the Serranidae [23] in the Philippines; eight species of the Ariidae [24] and 36 species of the Carangidae in the coast of Malaysia [25]; some species of genera *Amphiprion* and *Premnas* in Thailand [26] and 26 species of the Gobiidae in Vietnam [27]. Zhang and Hanner [15] conducted a relatively comprehensive survey of fish biodiversity in the South China Sea (SCS), with 242 species successfully identified by DNA barcoding from 1336 specimens. However, Zhang and Hanner’s survey mainly focused on the southern coast of China and the middle SCS. Peripheral countries such as Vietnam with long coastlines and various coastal landforms, which may contribute massive biodiversity, are not included in their study.

Vietnam’s coast has been drawing much attention due to its unique geographical location and marine environment characteristics which show physical, environmental and current gradients along its extraordinarily long coastline [28–31]. The various gradients could be potential influences on biodiversity and structure of fish community in northern, central and southern areas, and variations in fish community could be the biological basis for fishery management in Vietnam. At least 2083 marine fish species belonging to 717 genera and 198 families have been recorded in Vietnam [32, 33], and among which 130 fish species are considered economically important species [34]. Fishery resources play an important role in the economy of Vietnam [35], and the fisheries are a major source of food and have helped coastal communities to maintain their livelihoods and community structure. However, the coastal ichthyofaunae of Vietnam remain unclear due to lack of taxonomists [36] whose training is time-consuming and species delimitation requires painstaking labor. Hence, application of suitable methods for accurate and quick identification of fish will be crucial to assist in managing fisheries for long-term sustainability, and will improve ecosystem research and conservation.

DNA barcoding would be a powerful tool to improve the knowledge of fish fauna in Vietnam. However, there have been only a few DNA barcoding studies of fishes in Vietnam and most of them were focused on a small taxonomic scale and/or samples collected from a limited area [37–40]. A comprehensive DNA barcoding study on the coastal fishes of Vietnam is urgently needed, especially before its’ growing fishery industry threatening the diversity. The present study is aimed (1) to DNA barcode fishes collected from Vietnam in order to provide a species list confirmed by *COI* sequences and (2) to preliminarily examine the faunae among fishes collected from northern, central and southern Vietnam.

## Material and methods

### Sample collection

**Fig 1:**
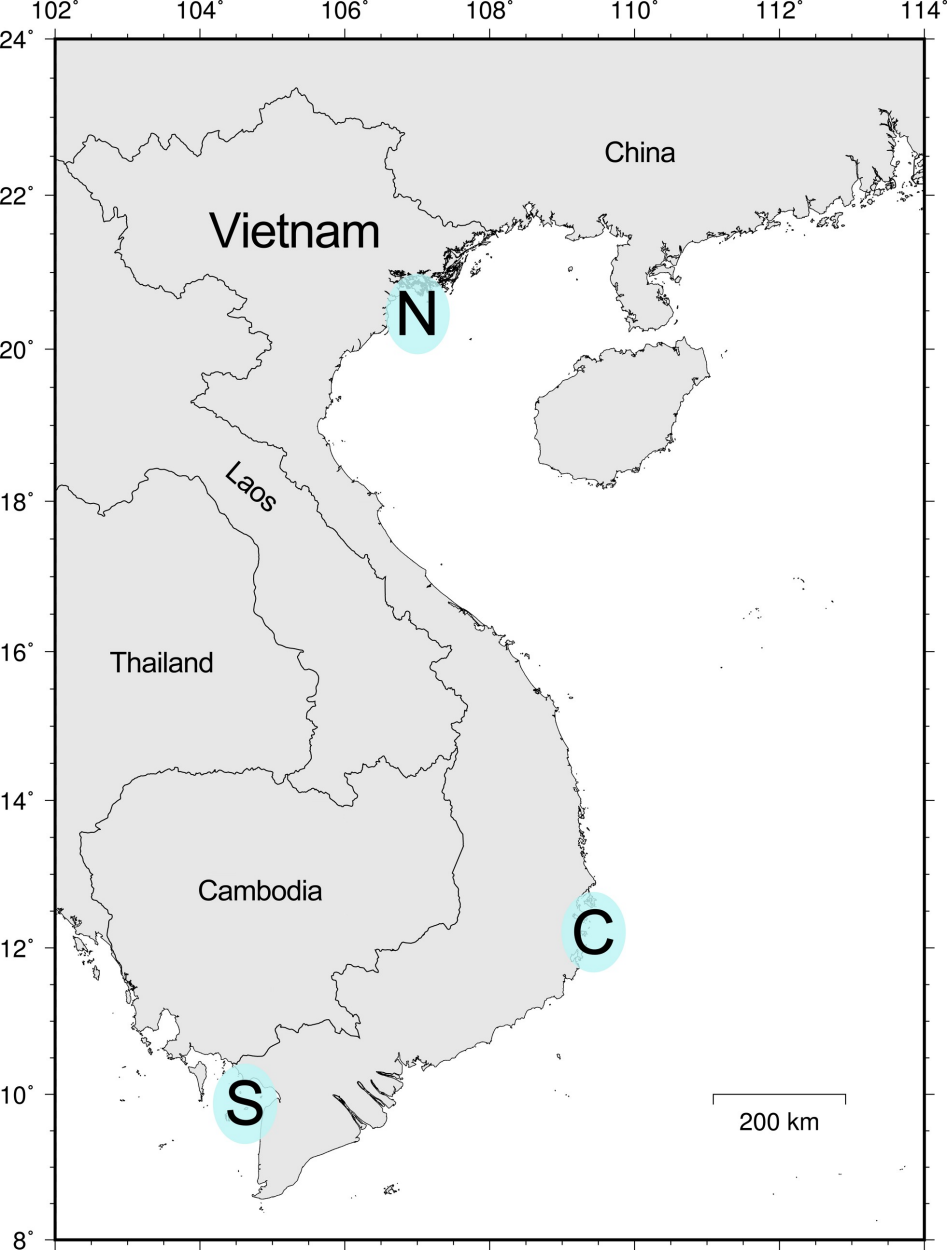
Three sampling areas along the coast of Vietnam. N comprises Do Son (20.719536, 106.793336) and Ha Long (20.948599, 107.083191) of Hai Phong–Quang Ninh Provinces; C comprises Phan Rang (11.591625, 109.046675; 11.546292, 109.025067) of Ninh Thuan Province; S comprises Tac Cau (9.876155, 105.124151) and Ha Tien (10.378664, 104.485162) of Kien Giang Province. GPS coordinates are for the major fish markets visited every day.

Through 2016 to 2017, fish specimens were collected from fish markets at five sampling sites that represented three geographic areas along the coast of Vietnam, including Do Son and Ha Long of Hai Phong–Quang Ninh Provinces (Northern area, abbreviated as N, 14 days of sampling effort), Phan Rang of Ninh Thuan Province (Central area, C, 34 days), and Tac Cau and Ha Tien of Kien Giang Province (Southern area, S, 15 days) (Fig 1). Each specimen was photographed, and fin clips or muscle tissues were cut and stored in 95% ethanol and frozen at − 20°C before DNA extraction. For those large specimens and/or expensive fishes, only tissue samples were preserved. Voucher specimens were fixed with 10% formalin and then transferred to 70% ethanol solution for preservation. Voucher specimens and tissue samples were transported to Taiwan and deposited in the Department of Oceanography, National Sun Yat-sen University, Kaohsiung (DOS).

### DNA sequencing

An initial species checklist was built based on morphological identification of photographs and specimens. One specimen of each species from each geographic area depending on morphological-based identification was selected to extract DNA for further molecular analysis. Crude DNA was extracted from the tissue samples using a GeneMark DNA Purification Kit (GMbiolab, Taichung, Taiwan). Polymerase chain reaction (PCR) was performed to amplify mitochondrial cytochrome *c* oxidase subunit I (*COI*) using primers designated by Ward, Zemlak (5]: FishF1 (5’-TCAACCAACCACAAAGACATTGGCAC-3’); FishF2 (5’-TCGACTAATCATAAAGATATCGGCAC-3’) and FishR1 (5’- TAGACTTCTGGGTGGCCAAAGAATCA-3’); FishR2 (5’-ACTTCAGGGTGACCGAAGAATCAGAA-3’). PCRs were run with a reaction volume of 25 μL containing 3 μL of template DNA (50 ng μL^-1^), 3 μL of 10x buffer, 2 μL of dNTPs (2.5 mM), 1.2 μL of each primer (10 μM), 0.13 μL of *ProTaq Plus* polymerase (Protech, Taipei) and 14.47 μL of deionized water. PCR was performed with an initial denaturation at 94°C for 5 min, followed by 35 cycles of amplification (denaturing at 94°C for 30 s, annealing at 50°C for 30 s, and extension at 70°C for 1 min) and a final extension at 72°C for 8 min. PCR products were checked by electrophoresis with 1.5% agarose gel and then purified by the SAP-Exo purification kit (Jena Bioscience, Jena) according to the manufacturer’s protocols. Sequences were generated by an ABI 3730 automated sequencer and edited manually using MEGA version X [41]. All sequences were translated into amino acids to confirm the effectiveness of the sequences and to detect the presence of nuclear DNA pseudogenes, insertions, deletions, or stop codons. All sequences were submitted to GenBank, and their accession numbers were listed in S1 Table while catalog numbers of tissue samples were included in the description of each sequence in the NCBI webpage. Voucher specimens and tissue samples have identical catalog numbers.

### Data analyses

DNA barcoding was conducted using *COI* sequences blasted in the GenBank and BOLD online databases with a threshold of similarity higher than 98% [42]. Specimens would be re-examined if any conflict exists between morphological and molecular identification. The final species list was compared with four literatures of Vietnamese fishes as well as the Fishbase online database to determine the number of new record species [43–46]. After blast in online database, *COI* sequences were aligned and trimmed to the same length using MEGA X for subsequent analysis. The Kimura-2-Parameter (K2P) genetic distances [47] were calculated at different taxonomic levels, including intraspecific, interspecific within the same genus, inter-generic within the same family, inter-family within the same order, and inter-order in the same class. For intraspecific level, species that only contain one sequence were excluded from the distance analysis; for intra-generic level, genera that contain only one species were excluded, and this criterion was applied for the higher levels of genetic distance analysis. Genetic distances at all levels were calculated disregarding the source of the specimens. The values may contain intra- and/or inter-regional divergences. According to Ward [48], species that shows intraspecific variations above 2% were treated as different species. Higher taxonomy, order and family, in the present study followed Betancur-R et al. [49]. A neighbour-joining (NJ) tree of all analyzed *COI* sequences was built using Tamura-Nei + Γ model recommended by MEGA X [50] with 1,000 bootstrap replications [51]. Both model test and tree construction were conducted by MEGA X.

## Results

A total of 3,638 tissue samples were collected from Vietnam, among which 765 *COI* sequences were obtained (Table 1). Base on morphological and molecular identifications, these samples represented 458 species of 273 genera, 113 families and 43 orders (S1 Table). Among the 458 species, 59 fishes were new records in Vietnam, sequences of six species were new to the Genbank and BOLD online databases, and only 32 species cannot be annotated to species level because difficulty in morphological identifications and their genetic distances to most similar sequences were more than 2% (S1 Table). The length of all barcode sequences ranged from 313 to 683 bp, with an average of 588 bp and 81% of sequences were longer than 550 bp. No stop codon, insertion, or deletion was observed in any of the obtained sequences.

**Table 1:** Summary of specimens, sequences and species number in each area in this study.

Areas	No. of specimens	No. of sequences	No. of species	No. of species used in analyses* (707 sequences)
Northern	896	191	135	129
Central	1741	359	274	256
Southern	1001	215	161	159
Total	3638	765	458	444

*Sequences from [27] are included here and those sequences shorter than 500 bp are not included in the analyses.

**Fig 2:**
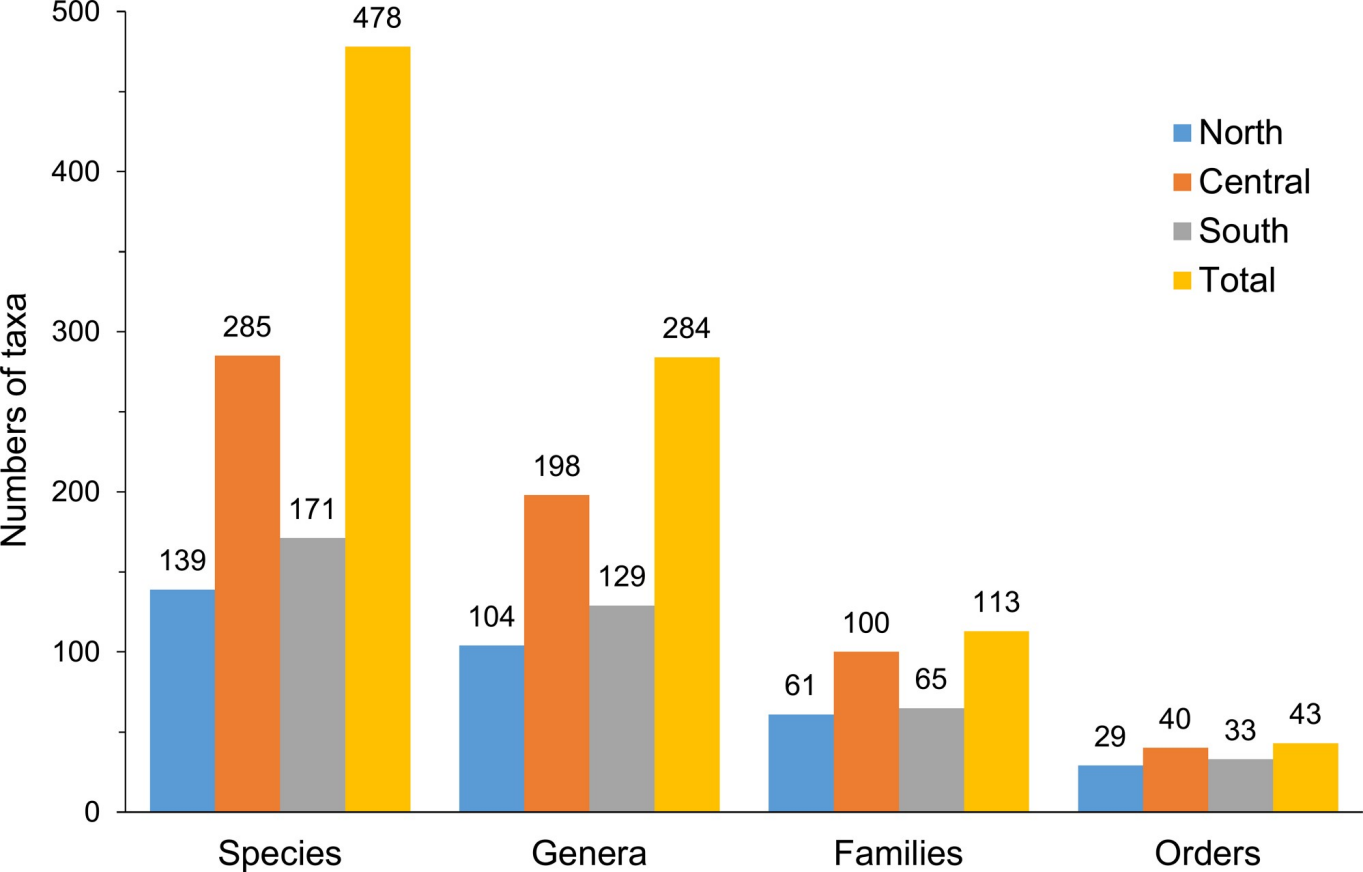
Variation of taxonomic levels in each area. Data include records from [27].

Thirty specimens and their *COI* sequences from Nguyen et al. [27] were included and re-identified in this study. In total, there were 795 *COI* sequences of 478 species, 284 genera, 113 families and 43 orders for the tree reconstruction (S1 Table and Fig 2). After alignment, all sequences were trimmed to 500 bp and those shorted than 500 bp were abandoned. In total, 707 sequences, including those not able to be determined to species level, were remained for subsequent analyses (444 species of 264 genera, 105 families and 42 orders).

**Fig 3:**
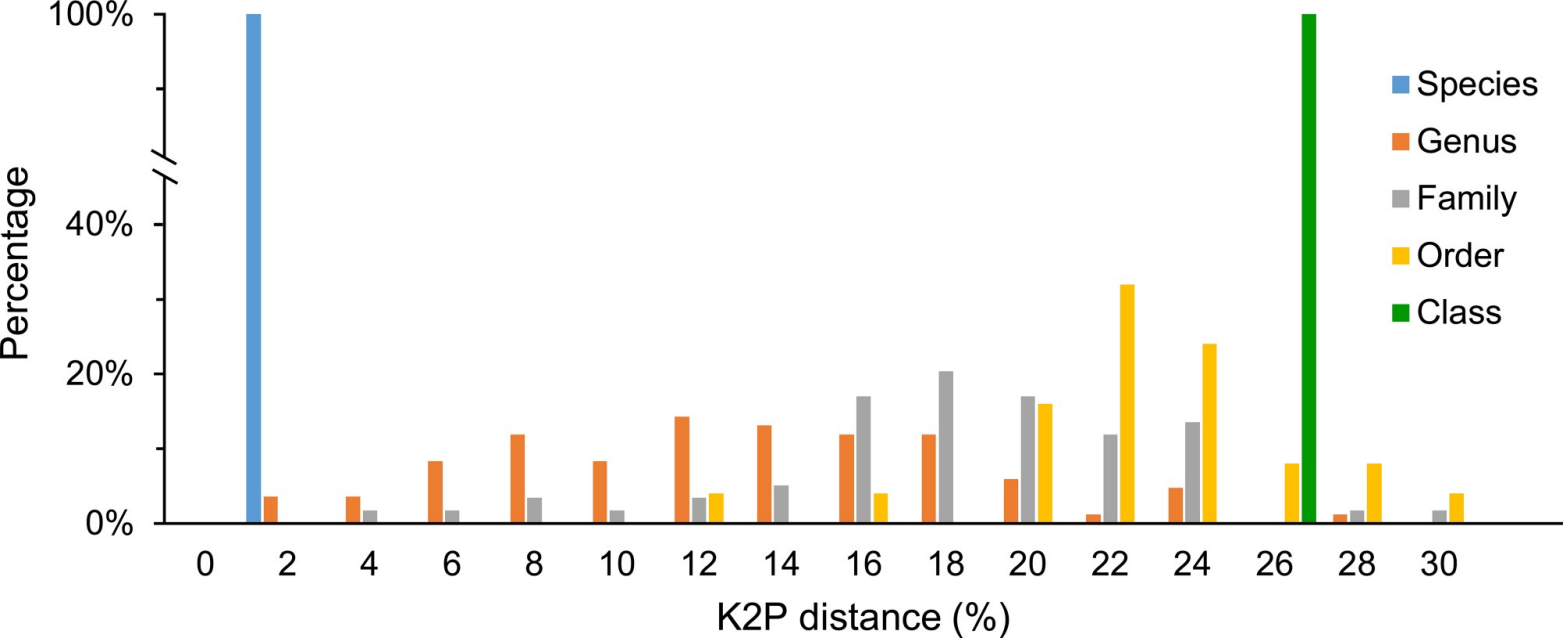
Distribution of K2P distances (percent) for mitochondrial *COI* (707 sequences, 500 bp) at different taxonomic levels. Data include records from [27].

The K2P genetic distances within each taxonomic level are summarized in Table 2. The average intraspecific K2P genetic distance was 0.34 ± 0.03%. Intra-generic, -families, -orders, and -class genetic distances were 12.14 ± 0.61%, 17.39 ± 0.63%, 21.42 ± 0.72% and 24.80 ± 1.70%, respectively (Table 2). An increase in genetic variation at higher taxonomic levels was observed, but the rate of increase declined with the higher taxonomic categories (Fig 3). K2P genetic distances of 10 species (*Platycephalus indicus*, *Fistularia petimba*, *Plotosus lineatus*, *Uroconger lepturus*, *Trypauchen vagina*, *Scolopsis vosmeri*, *Scatophagus argus*, *Seriolina nigrofasciata*, *Ablennes hians*, *Decapterus maruadsi*) were greater than 2% (Table 3) and their sequences were divided into two clades. However, despite the high genetic distance, all their sequences were identified as one same species in BLAST results. In order to validate the sequences of these 10 species, additional *COI* sequences of each species were obtained from BOLD and analysed together with the sequences generated in the present study. The NJ trees with selected outgroups (S1 Fig) showed that each of those species can be separated into different clusters.

**Table 2:** Summary of genetic divergences (K2P percent) within various taxonomic levels based on 707 *COI* sequences.

Comparisons within	Taxa	K2P distances (%)	
		Range	Mean ± SE
Species	142	0.00–1.42	0.34 ± 0.03
Genus	84	1.44–26.05	12.14 ± 0.61
Family	59	3.92–28.03	17.39 ± 0.63
Order	25	10.92–28.03	21.42 ± 0.72
Class	1	-	24.80 ± 1.70

**Table 3:** Summary of K2P genetic divergences of 10 species with intraspecific distances more than 2%.

Species	K2P distance (%)	Area	Compared sequences
*Platycephalus indicus*	16.3 ± 2.0	N vs. N	2
*Fistularia petimba*	12.0 ± 1.8	C, S vs. C	6
*Plotosus lineatus*	11.7 ± 1.6	N, C vs. S	3
*Uroconger lepturus*	10.7 ± 1.5	C vs. C	3
*Trypauchen vagina*	10.4 ± 1.5	N vs. S	3
*Scolopsis vosmeri*	10.1 ± 1.4	N, C vs. S	4
*Scatophagus argus*	7.5 ± 1.3	N vs. S	2
*Seriolina nigrofasciata*	3.6 ± 0.8	C, S vs. C	3
*Ablennes hians*	2.4 ± 0.7	N vs. C, S	3
*Decapterus maruadsi*	2.3 ± 0.7	C vs. S	3

N–Northern Vietnam; C–Central Vietnam; S–Southern Vietnam

The NJ tree based on 707 *COI* fragments showed that all sequences from the same species were monophyletic (10 aforementioned species with large intraspecific distances were treated as different species; S2 Fig). At the intra-generic level, however, 36 genera were not monophyletic, including *Acanthocepola*, *Acentrogobius*, *Alepes*, *Ambassis*, *Arnoglossus*, *Callionymus*, *Carangoides*, *Chaetodon*, *Chelon*, *Cynoglossus*, *Evynnis*, *Gerres*, *Gymnothorax*, *Hyporhamphus*, *Leiognathus*, *Lethrinus*, *Lutjanus*, *Ostorhinchus*, *Paramonacanthus*, *Parupeneus*, *Pentapodus*, *Priacanthus*, *Pseudorhombus*, *Rogadius*, *Sardinella*, *Saurida*, *Scorpaenopsis*, *Secutor*, *Sillago*, *Sphyraena*, *Stolephorus*, *Strongylura*, *Synodus*, *Taeniamia*, *Terapon*, *Zebrias*.

Only 16 out of 478 species were collected from all three areas (S1 Table). Numbers of common species are much higher between areas, with 45, 48 and 40 for N-C, C-S and N-S, respectively (Table 4). The numbers of common species show a trend with decreased distances.

**Table 4:** Similarity of species composition among areas.

Between groups	N–C	C–S	N–S	N–C–S
Common species/total	45/379	48/408	40/270	16/478
Similarity (%)	11.87	11.76	14.81	3.35

Data include records from Nguyen et al. [27].

## Discussion

As in previous research [5, 6, 12–19, 22–26, 52], DNA barcoding analysis based on the *COI* gene accompanying morphological identifications was able to identify most fishes to the species level (446 out of 478) in the coast of Vietnam with limited similarity in fish fauna among our sampling areas (S1 Table). A reliable DNA barcode reference library for the coastal fish in Vietnam was established, and it would facilitate future studies in taxonomy, conservation, fishery management and so on by linking barcode sequences with carefully identified voucher specimens. However, since 32 species cannot be annotated to species level, it implies that more efforts are needed to sequence *COI* fragments in order to enrich the databases.

Although it is highly controversial [53], the distance-based technique remains as the standard approach in DNA barcoding [54]. In this study, the K2P model was used to ensure consistency and comparability with other barcoding studies. These results indicate that using *COI* gene sequences as DNA barcodes to discriminate fish species in Vietnam is feasible. Increasing average genetic distance values were obtained at higher taxonomic levels in this study (Fig 3). The average genetic distances between individuals within species, genera, families and orders were 0.34%, 12.14%, 17.39% and 21.42%, respectively, consistent with the patterns observed in other fish barcoding studies. For example, the K2P values within species, genera, families, and orders were 0.39%, 9.93%, 15.46%, and 22.18% in Australia [5]; 0.30%, 6.60%, 9.91%, and 16.00% in India [18]; 0.18%, 13.55%, 19.65%, and 24.05% in SCS [14]; 0.21%, 5.28%, 21.3%, and 23.63% in Rongcheng Bay, China [17]. The intraspecific divergence (about 0.34%) was much smaller than 2% in Vietnam, but the much lower intraspecific divergence is also observed in previous studies (0.3% in Lakra et al. [18]; 0.319% in Zhang, 2011 [55]; 0.18% in Zhang and Hanner [15]; 0.37% in Jaafar et al. [25]; 0.21% in Bingpeng et al. [16]). It is worth mentioning that sequences used for analyses in this study only with a length of 500 bp while all references mentioned above used the lengths between 605–655 bp, indicating the sufficient effectiveness even though using shorter sequences.

In a total of 478 fish species obtained from 795 *COI* sequences, there were 139, 285 and 171 species in northern, central and southern areas, respectively. In addition to species diversity, highest numbers of all taxonomic levels were observed in central Vietnam (Fig 2). The highest diversity may be due to the longer sampling period (34 vs. 14 and 15 days in the north and south, respectively), and sampling seasons may also be a concern (Dec., June, and Sep. for north, central and south, respectively). In addition, habitat diversity may also contribute to the fish fauna biodiversity. Most substrates in the northern and southern Vietnam are sandy and muddy while the central part of Vietnam contains various landforms of rocks and coral reefs in addition to sandy substrates. Habitat diversity may result in the higher fish biodiversity in central Vietnam.

There was a huge difference in species composition among geographic areas, only 16 out of 478 species were collected from all three areas (S1 Table). Numbers of common species are much higher between areas, with 45, 48 and 40 for N-C, C-S and N-S, respectively (Table 4). Since the geological distance of C-S is the shortest among three areas, the numbers of common species show a trend with decreased distances. However, variation in physical and environmental gradients may also be associated with the trend along the coastline over 3,260 km [28]. Our northern and southern sampling areas located at nearly the distal ends of the Vietnamese coastline, and it has been reported that the coast of Vietnam exhibits a remarkable environmental shift from north to south. The environmental gradients include average sea surface temperature (23–26–27°C or 25–28–30°C depending on studies); salinity (31–34–31 psu), and dissolved oxygen (4.5–4.0–3.8 ml L^-1^) [29, 30]. These physical factors are definitely important influences shaping the fish faunae. Moreover, the prevailing currents along the coast of Vietnam are also divided into three segments, approximately corresponding to our sampling sites [31]. The middle segment off the central Vietnamese coast may have a meandering northward coastal current and cyclonic gyre in different seasons while the north and south segments are rather stable in current directions [31]. Since currents may influence pelagic larval dispersal, complex currents might raise biodiversity of coastal species as shown in our central area.

Only 98 out of 478 species (20.50%) in this study have been repeatedly observed in Zhang and Hanner [15]. Numbers of common species between each area in this study and Zhang and Hanner [15] are 58 out of 139 (41.73%) in N, 46 out of 285 (16.14%) in C, and 46 out of 171 (26.90%) in S. The species composition in N is most similar to Zhang and Hanner’s study in SCS. Most sampling of Zhang and Hanner’s survey were carried out along the southern coast of China, and results in higher number of common species due to closer geological distance as shown in C-S. Besides, our sampling sites in each area were mainly focused on fish landing sites that most of fishes were obtained from the nearshore area. This may partially explain why species composition of N is rather similar to that of Zhang and Hanner [15] in SCS.

In the NJ tree of 707 DNA barcode sequences, monophyly occurred in every single species but not in each genus. This result indicates that the DNA barcode is reliable in diagnosis at species level but the resolution is not good enough for determining higher taxonomic levels (e.g. genus and family). There are 36 genera not monophyletic in the NJ tree. We used counterpart sequences in the same family from BOLD to reconstruct additional trees for each non-monophyletic genus (data not shown). Most non-monophyletic genera can be monophyletic in additional trees that only contain sequences from the same family except for eight genera (*Acentrogobius*, *Chaetodon*, *Gymnothorax*, *Leiognathus*, *Lutjanus*, *Secutor*, *Sillago* and *Stolephorus*), implying the needs of further taxonomic revisions. The genus *Acentrogobius* of the Gobiidae is paraphyletic nested by *Parachaeturichthys*. The paraphyly is consistent with the *COI* tree in Lavery Clements [56], and further studies may be necessary to clarify their relationship. *Chaetodon* of the Chaetodontidae is paraphyletic nested by *Parachaetodon*, which has been considered a synonym of *Chaetodon* [57]. The largest genus *Gymnothorax* in the Muraenidae is polyphyletic, consistent with the mitochondrial study using *12S* and *16S* genes [58]. *Lutjanus* of the Lutjanidae is a paraphyletic group nested by *Caesio*, *Dipterygonotus* and *Pterocaesio*, three genera used to be placed in the Caesionidae but now in the Lutjanidae [49]. The result is consistent with the relationships shown in Guo [59] based on complete mitochondrial genomes. *Leiognathus* and *Secutor* of the Leiognathidae are polyphyletic in our additional tree. *Leiognathus* has been revealed as a paraphyletic genus in Ikejima [60]. However, *Secutor* was monophyletic according to Zhang and Hanner [15]. The ambiguous relationship among genera in the Leiognathidae may be attributed to massive misidentifications of previously uploaded sequences [15]. In the Sillaginidae, *Sillaginodes* is nested in *Sillago*. However, this result is opposite to the morphological tree reconstructed by Kaga [61]. Similar to the Leiognathidae, sequences of sillaginid fishes may contain misidentifications due to their highly similar morphologies. *Stolephorus* of the Engraulidae is polyphyletic in this study but monophyletic in Qu [62] reconstructed using complete mitochondrial genomes. The 500 bp length sequences may not provide sufficient information to separate *Stolephorus* from its counterparts.

## Conclusions

It is confirmed that DNA barcoding is efficient and reliable tools for identification of coastal fishes at the species level in Vietnam. In this study, 765 specimens from 458 different species of 273 genera, 113 families in 43 orders were DNA-barcoded. A total of 59 species were newly recorded in Vietnam. The average genetic distance using K2P analysis of individuals within species, genera, families, orders and classes were 0.34%, 12.14%, 17.39%, 21.42% and 24.80%, respectively. Further, establishment of a reliable *COI* barcode database of coastal fish fauna in Vietnam may serve as a reference library for accurate identification of fishes that may facilitate ichthyological research, including taxonomy, fishery, biodiversity management and so on. Presence of high degree of pair-wise intra-specific divergence among the individuals of some species was revealed, suggesting the presence of cryptic species and advocating the need for more comprehensive studies on fish fauna of Vietnam.

## Supporting information

S1 Fig Neighbor-joining trees based on *COI* sequences from 10 species (showed in Table 3) with intraspecific K2P distances greater than 2%. Best chosen models were used with 1,000 bootstrap replications for tree reconstructions.
(PDF)

S2 Fig Neighbor-joining tree based on 707 *COI* sequences using Tamura-Nei + Γ model with 1,000 bootstrap replications. *and** denote 10 species with intraspecific distances more than 2%.
(PDF)

S1 Table The list of fish species DNA barcoded in this study. NR–New recoded species in Vietnam; ND–Not yet determined; N, C, S–northern, central, and southern areas in Vietnam; *and** denote 10 species with intraspecific distances more than 2% (in Table 3); species in bold were distributed in all areas; ● species recorded in this study; ○ species recorded from Nguyen et al. [27]; numbers in parentheses represent total species excluding those recorded in Nguyen et al. [27].
(DOC)
