# Supplementary material for: DNA barcoding of coastal ray-finned fishes in Vietnam
Source: PLoS One. 2019 Sep 19;14(9):e0222631. doi: 10.1371/journal.pone.0222631 (PMC6752846; doi:10.1371/journal.pone.0222631)
Supplement: S1 Fig — Best chosen models were used with 1,000 bootstrap replications for tree reconstructions. (PDF) [file pone.0222631.s001.pdf]

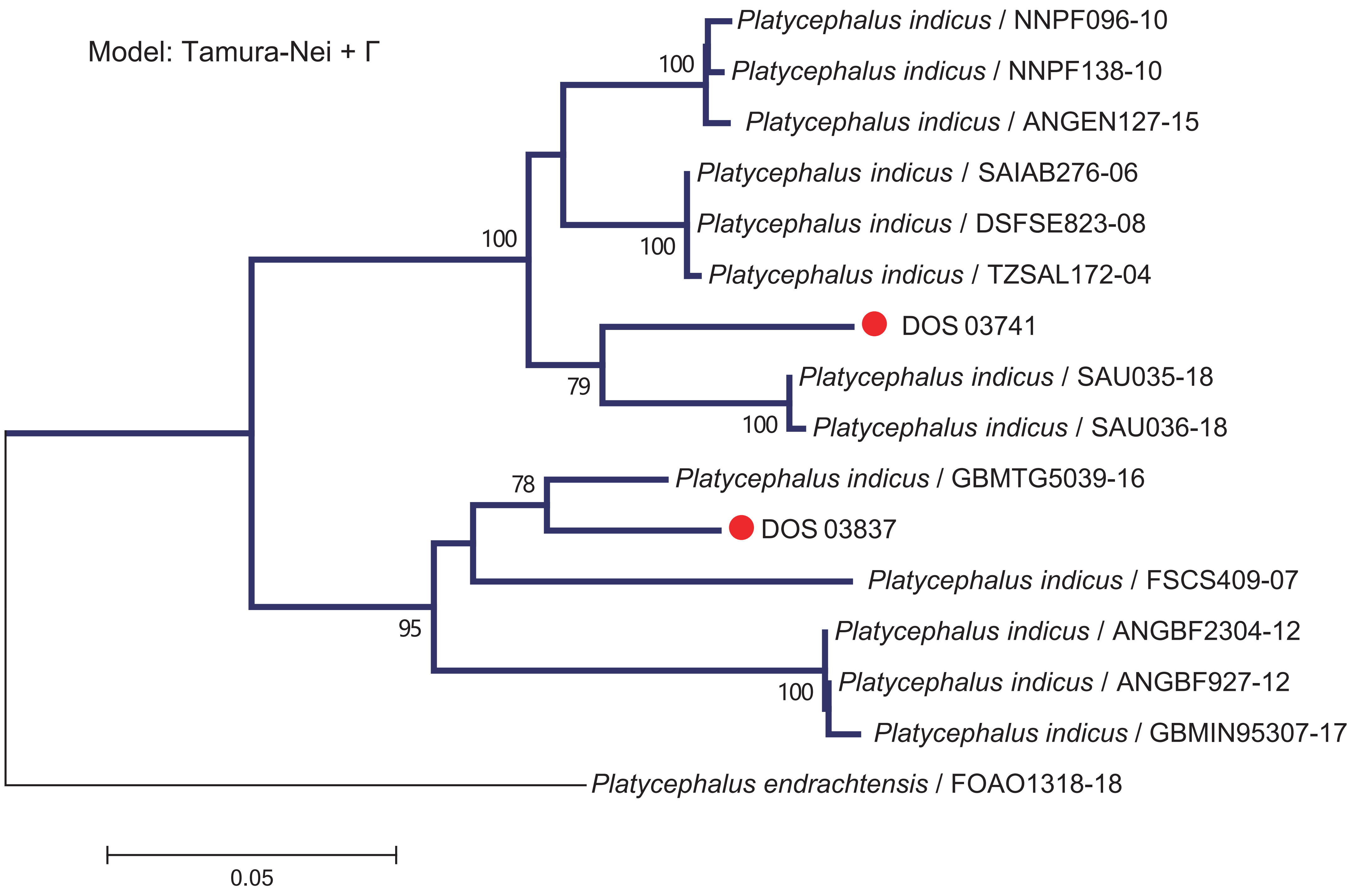

Model: Tamura-Nei + Γ
Platycephalus indicus / NNPF096-10
Platycephalus indicus / NNPF138-10
Platycephalus indicus / ANGEN127-15
Platycephalus indicus / SAIAB276-06
Platycephalus indicus / DSFSE823-08
Platycephalus indicus / TZSAL172-04
DOS 03741
Platycephalus indicus / SAU035-18
Platycephalus indicus / SAU036-18
Platycephalus indicus / GBMTG5039-16
DOS 03837
Platycephalus indicus / FSCS409-07
Platycephalus indicus / ANGBF2304-12
Platycephalus indicus / ANGBF927-12
Platycephalus indicus / GBMIN95307-17
Platycephalus endrachtensis / FOAO1318-18
100
100
100
79
100
78
95
100
0.05

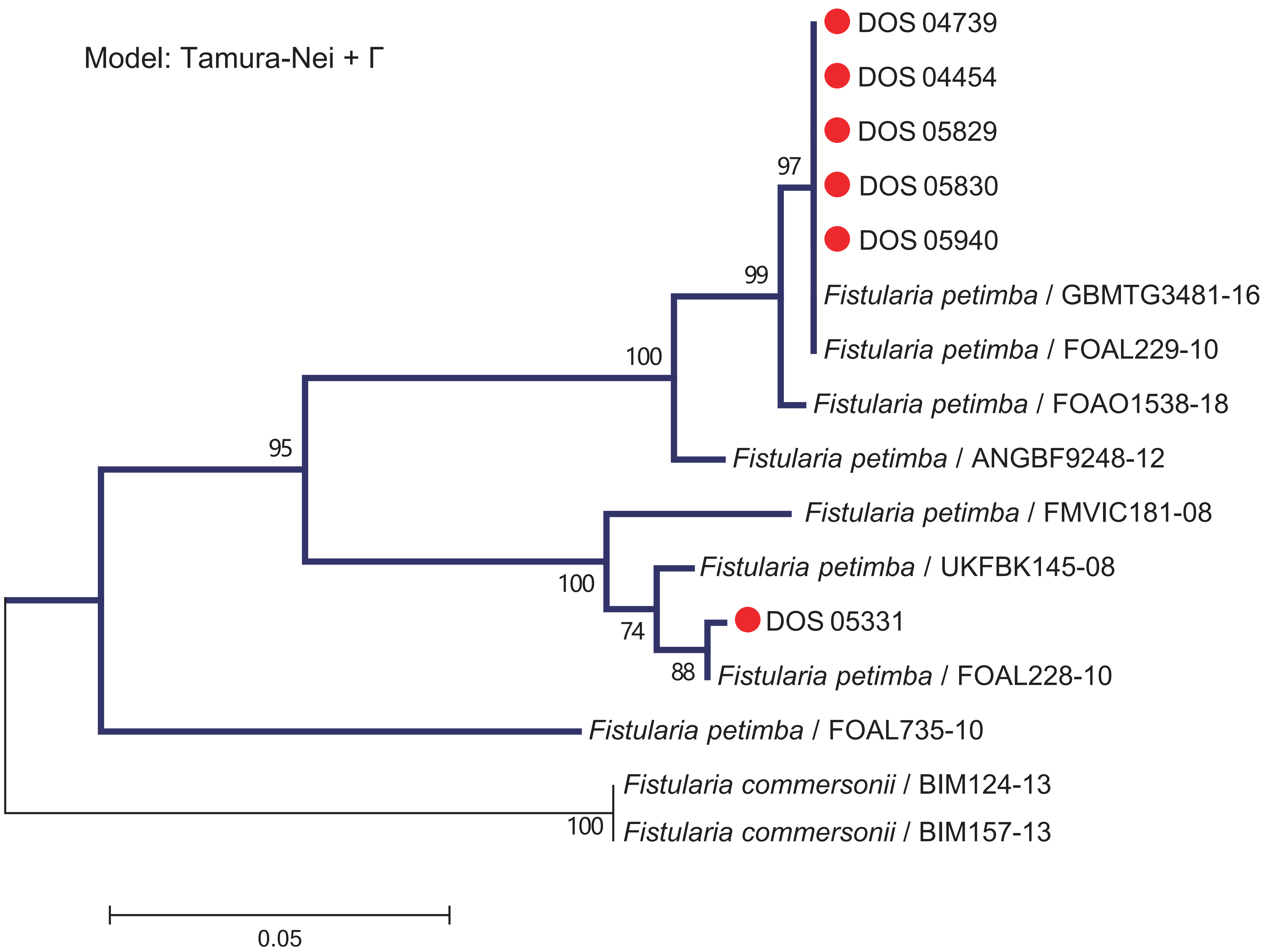
Model: Tamura-Nei + Γ
DOS 04739
DOS 04454
DOS 05829
DOS 05830
DOS 05940
Fistularia petimba / GBMTG3481-16
Fistularia petimba / FOAL229-10
Fistularia petimba / FOAO1538-18
Fistularia petimba / ANGBF9248-12
Fistularia petimba / FMVIC181-08
Fistularia petimba / UKFBK145-08
DOS 05331
Fistularia petimba / FOAL228-10
Fistularia petimba / FOAL735-10
Fistularia commersonii / BIM124-13
Fistularia commersonii / BIM157-13
97
99
100
95
100
74
88
100
0.05

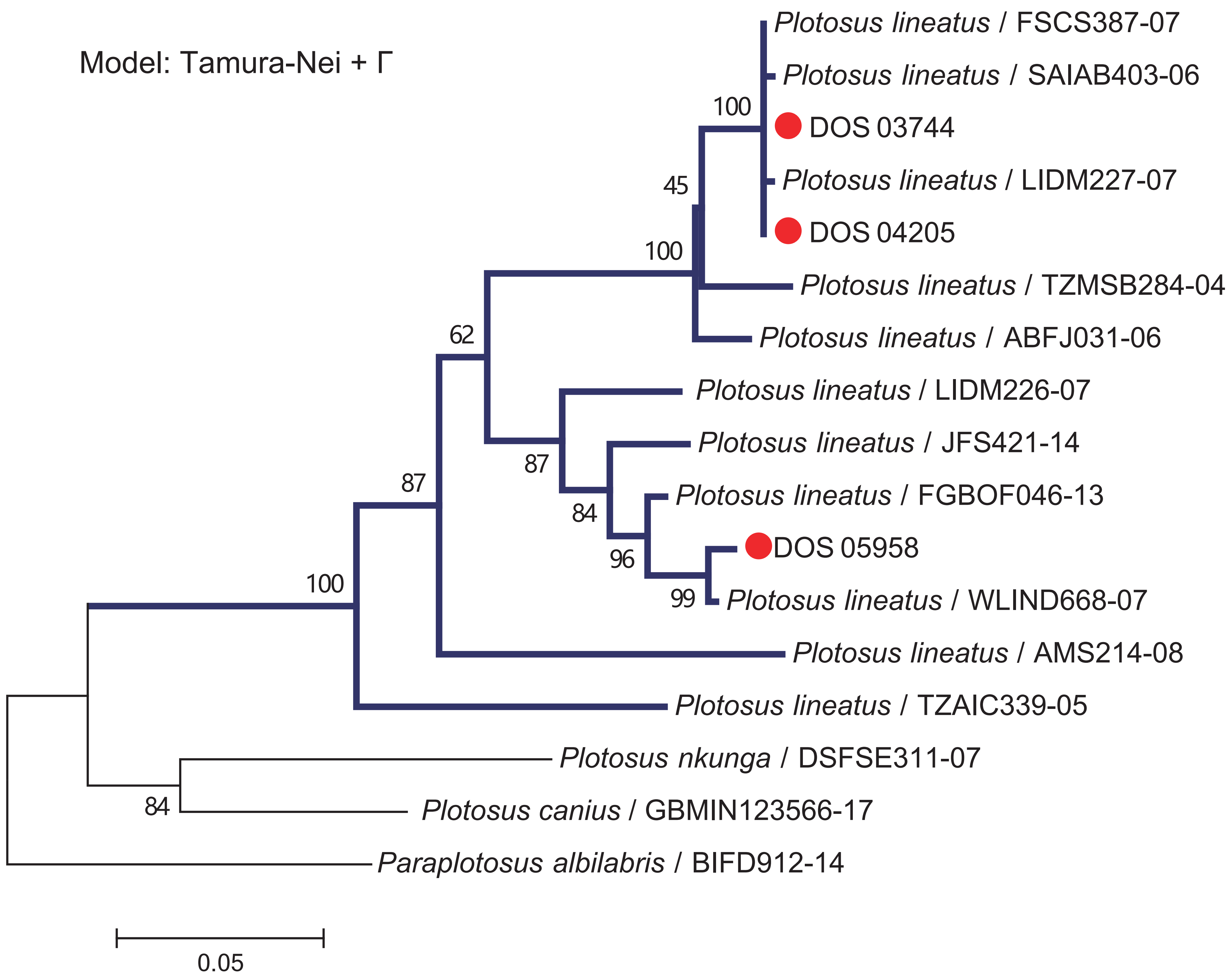

Model: Tamura-Nei + Γ
Plotosus lineatus / FSCS387-07
Plotosus lineatus / SAIAB403-06
DOS 03744
Plotosus lineatus / LIDM227-07
DOS 04205
Plotosus lineatus / TZMSB284-04
Plotosus lineatus / ABFJ031-06
Plotosus lineatus / LIDM226-07
Plotosus lineatus / JFS421-14
Plotosus lineatus / FGBOF046-13
DOS 05958
Plotosus lineatus / WLIND668-07
Plotosus lineatus / AMS214-08
Plotosus lineatus / TZAIC339-05
Plotosus nkunga / DSFSE311-07
Plotosus canius / GBMIN123566-17
Paraplotosus albilabris / BIFD912-14
100
45
100
62
87
84
96
99
87
100
84
0.05

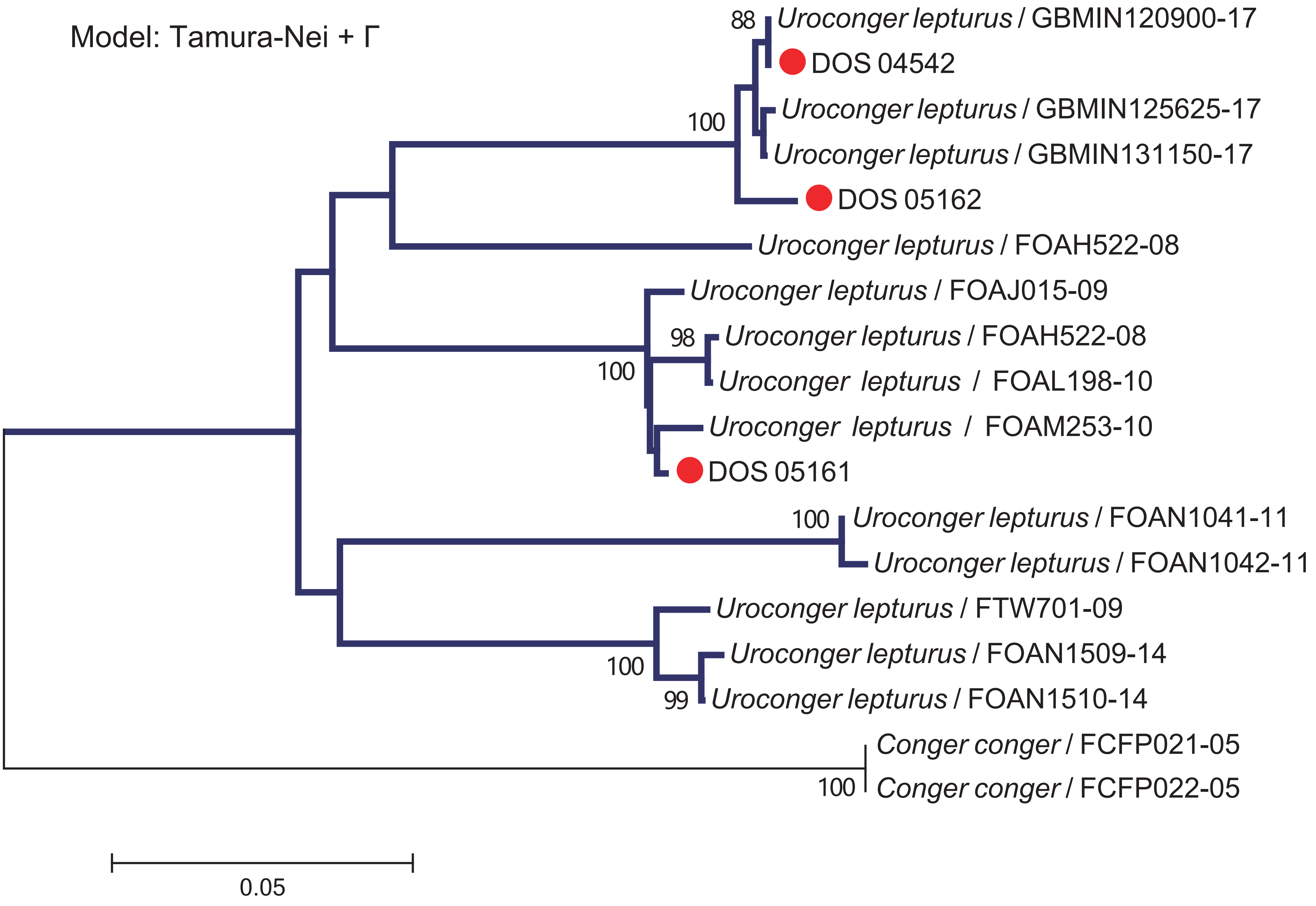

Model: Tamura-Nei + Γ
88
Uroconger lepturus / GBMIN120900-17
DOS 04542
100
Uroconger lepturus / GBMIN125625-17
Uroconger lepturus / GBMIN131150-17
DOS 05162
Uroconger lepturus / FOAH522-08
Uroconger lepturus / FOAJ015-09
98
Uroconger lepturus / FOAH522-08
100
Uroconger lepturus / FOAL198-10
Uroconger lepturus / FOAM253-10
DOS 05161
100
Uroconger lepturus / FOAN1041-11
Uroconger lepturus / FOAN1042-11
Uroconger lepturus / FTW701-09
100
Uroconger lepturus / FOAN1509-14
99
Uroconger lepturus / FOAN1510-14
Conger conger / FCFP021-05
100
Conger conger / FCFP022-05
0.05

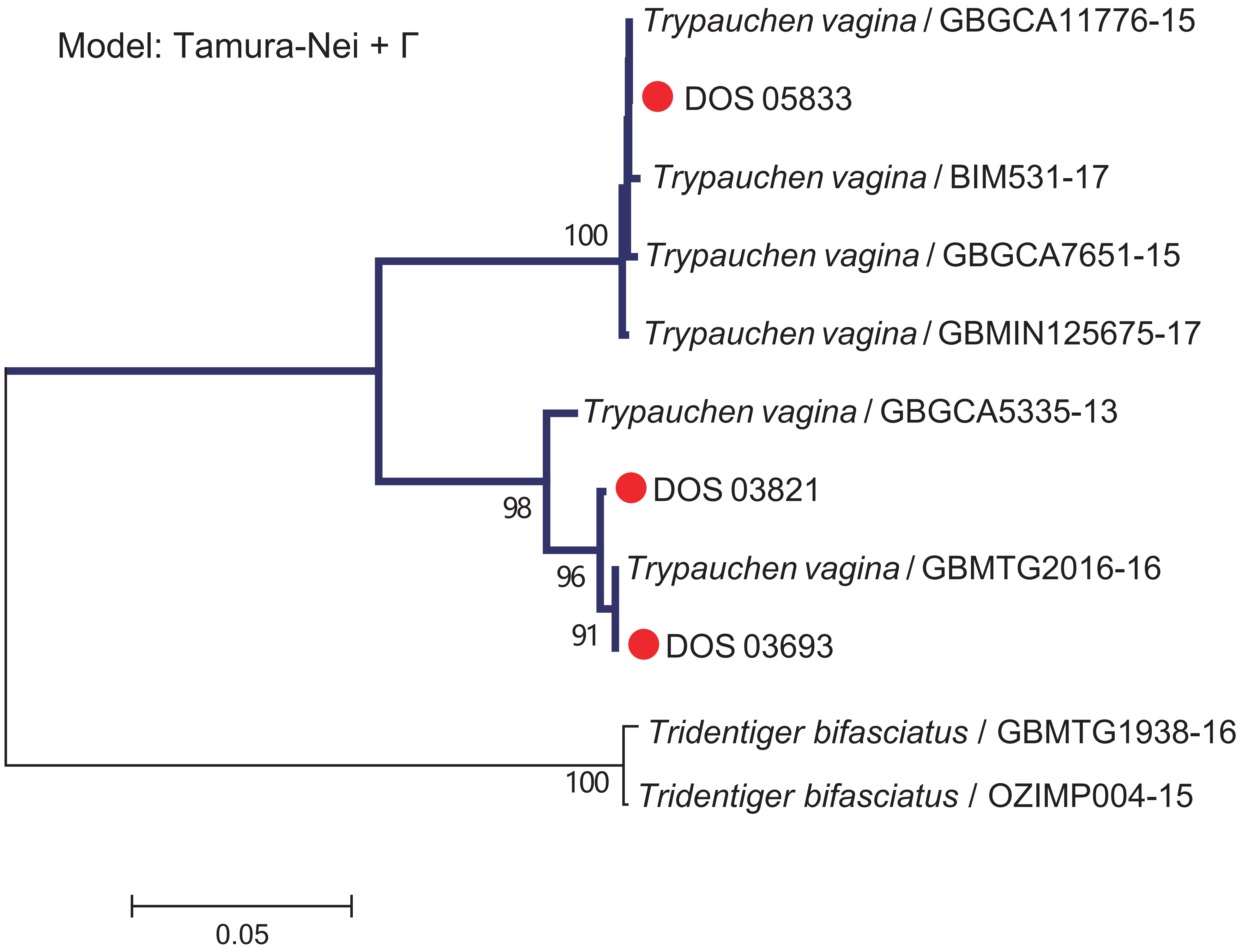

Model: Tamura-Nei + Γ
Trypauchen vagina / GBGCA11776-15
DOS 05833
Trypauchen vagina / BIM531-17
Trypauchen vagina / GBGCA7651-15
Trypauchen vagina / GBMIN125675-17
100
Trypauchen vagina / GBGCA5335-13
DOS 03821
98
Trypauchen vagina / GBMTG2016-16
96
91
DOS 03693
Tridentiger bifasciatus / GBMTG1938-16
100
Tridentiger bifasciatus / OZIMP004-15
0.05

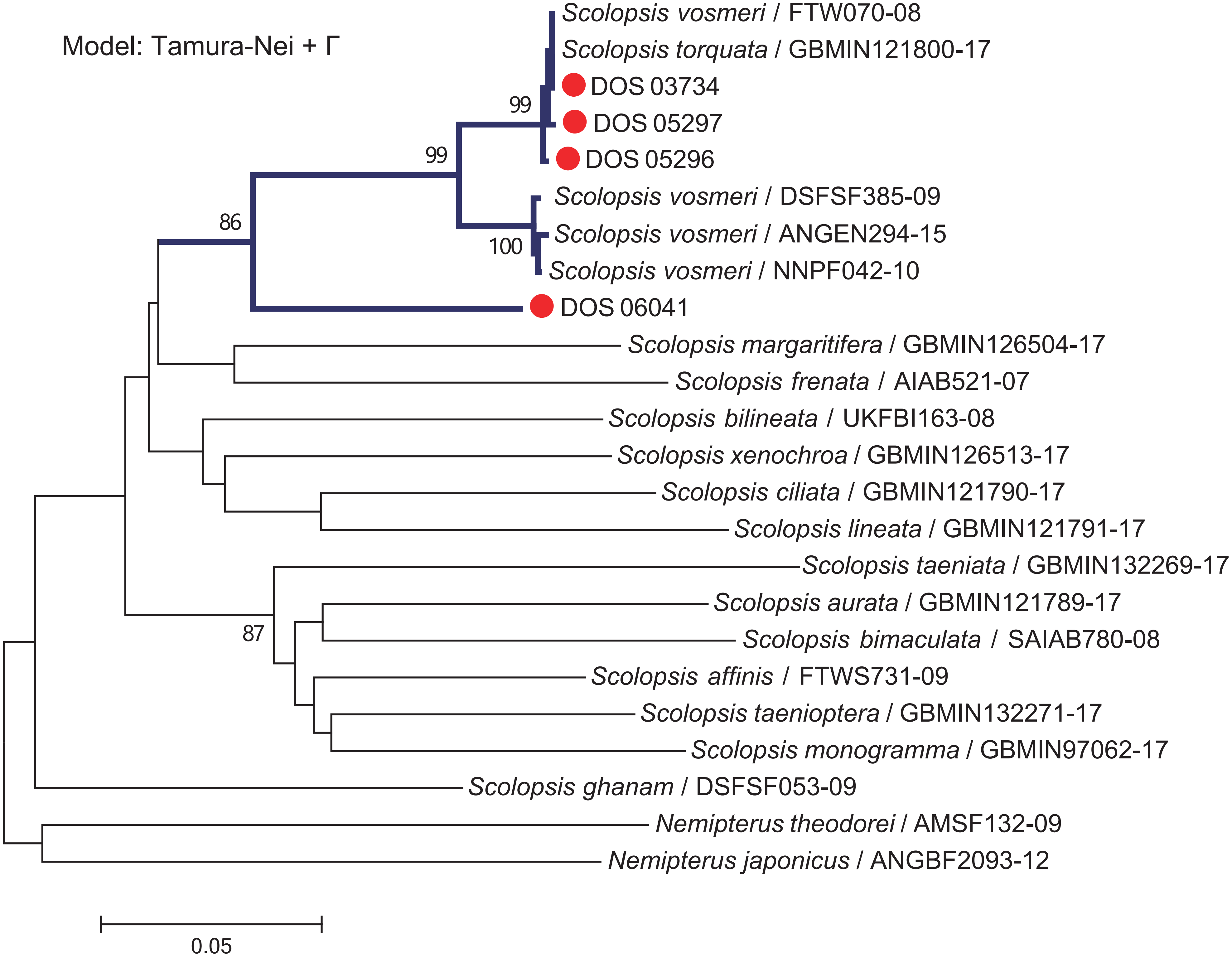
Model: Tamura-Nei + Γ
Scolopsis vosmeri / FTW070-08
Scolopsis torquata / GBMIN121800-17
DOS 03734
DOS 05297
DOS 05296
Scolopsis vosmeri / DSFSF385-09
Scolopsis vosmeri / ANGEN294-15
Scolopsis vosmeri / NNPF042-10
DOS 06041
Scolopsis margaritifera / GBMIN126504-17
Scolopsis frenata / AIAB521-07
Scolopsis bilineata / UKFBI163-08
Scolopsis xenochroa / GBMIN126513-17
Scolopsis ciliata / GBMIN121790-17
Scolopsis lineata / GBMIN121791-17
Scolopsis taeniata / GBMIN132269-17
Scolopsis aurata / GBMIN121789-17
Scolopsis bimaculata / SAIAB780-08
Scolopsis affinis / FTWS731-09
Scolopsis taenioptera / GBMIN132271-17
Scolopsis monogramma / GBMIN97062-17
Scolopsis ghanam / DSFSF053-09
Nemipterus theodorei / AMSF132-09
Nemipterus japonicus / ANGBF2093-12
99
99
86
100
87
0.05

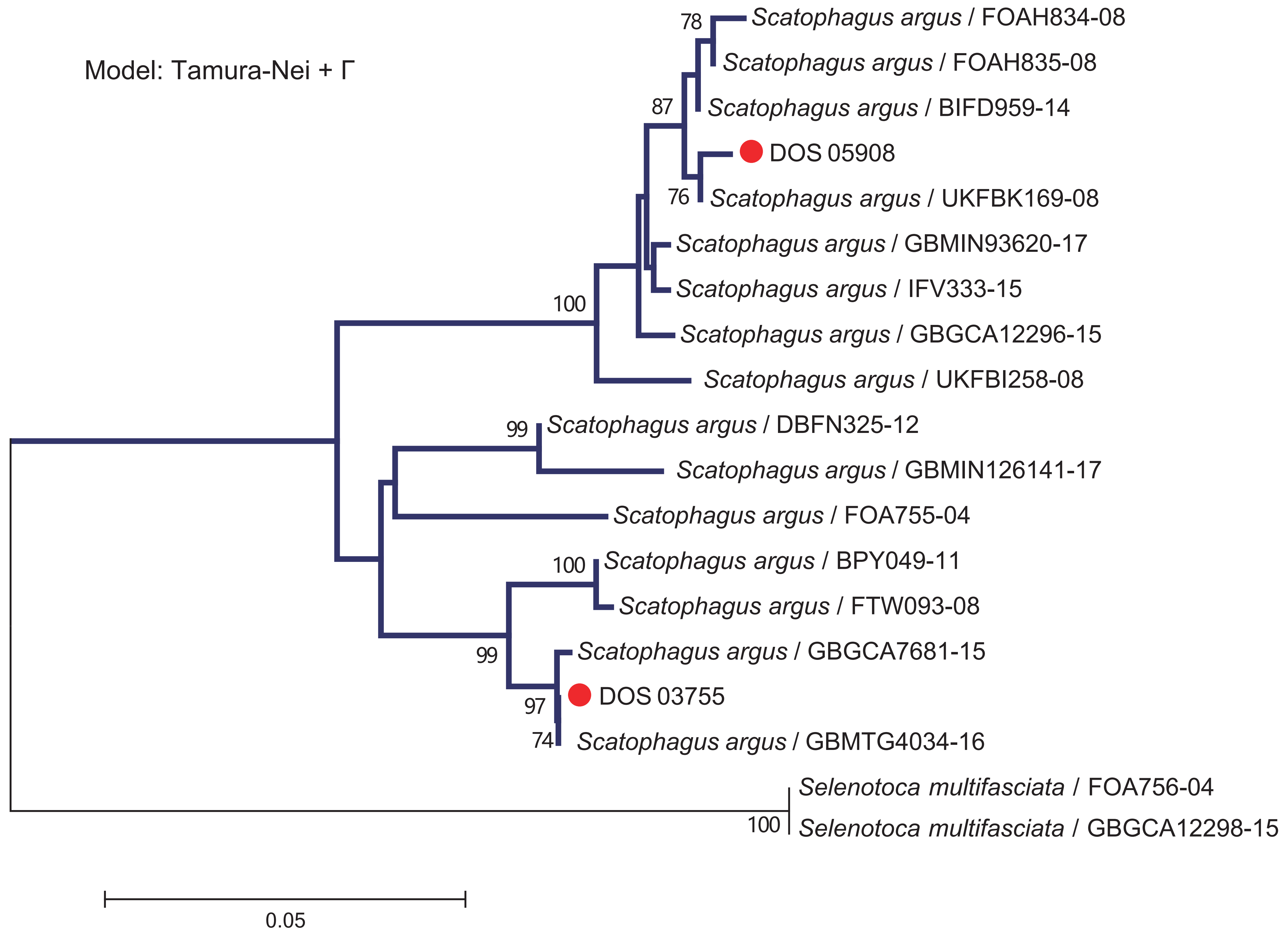

Model: Tamura-Nei + Γ
78
Scatophagus argus / FOAH834-08
Scatophagus argus / FOAH835-08
87
Scatophagus argus / BIFD959-14
DOS 05908
76
Scatophagus argus / UKFBK169-08
Scatophagus argus / GBMIN93620-17
Scatophagus argus / IFV333-15
100
Scatophagus argus / GBGCA12296-15
Scatophagus argus / UKFBI258-08
99
Scatophagus argus / DBFN325-12
Scatophagus argus / GBMIN126141-17
Scatophagus argus / FOA755-04
100
Scatophagus argus / BPY049-11
Scatophagus argus / FTW093-08
99
Scatophagus argus / GBGCA7681-15
DOS 03755
97
74
Scatophagus argus / GBMTG4034-16
Selenotoca multifasciata / FOA756-04
100
Selenotoca multifasciata / GBGCA12298-15
0.05

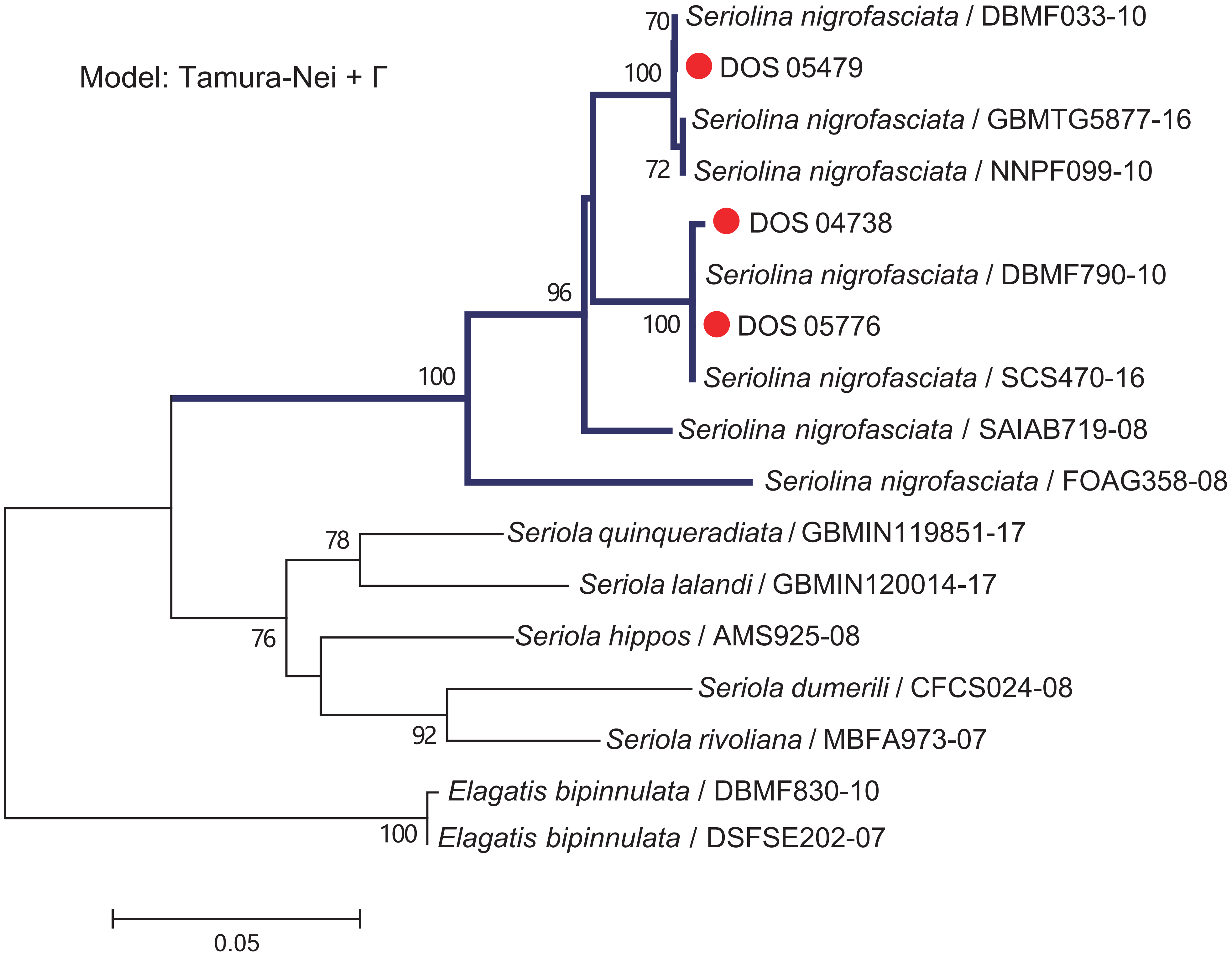

Model: Tamura-Nei + Γ
70
Seriolina nigrofasciata / DBMF033-10
100
DOS 05479
Seriolina nigrofasciata / GBMTG5877-16
72
Seriolina nigrofasciata / NNPF099-10
DOS 04738
96
Seriolina nigrofasciata / DBMF790-10
100
DOS 05776
100
Seriolina nigrofasciata / SCS470-16
Seriolina nigrofasciata / SAIAB719-08
Seriolina nigrofasciata / FOAG358-08
78
Seriola quinqueradiata / GBMIN119851-17
Seriola lalandi / GBMIN120014-17
76
Seriola hippos / AMS925-08
Seriola dumerili / CFCS024-08
92
Seriola rivoliana / MBFA973-07
Elagatis bipinnulata / DBMF830-10
100
Elagatis bipinnulata / DSFSE202-07
0.05

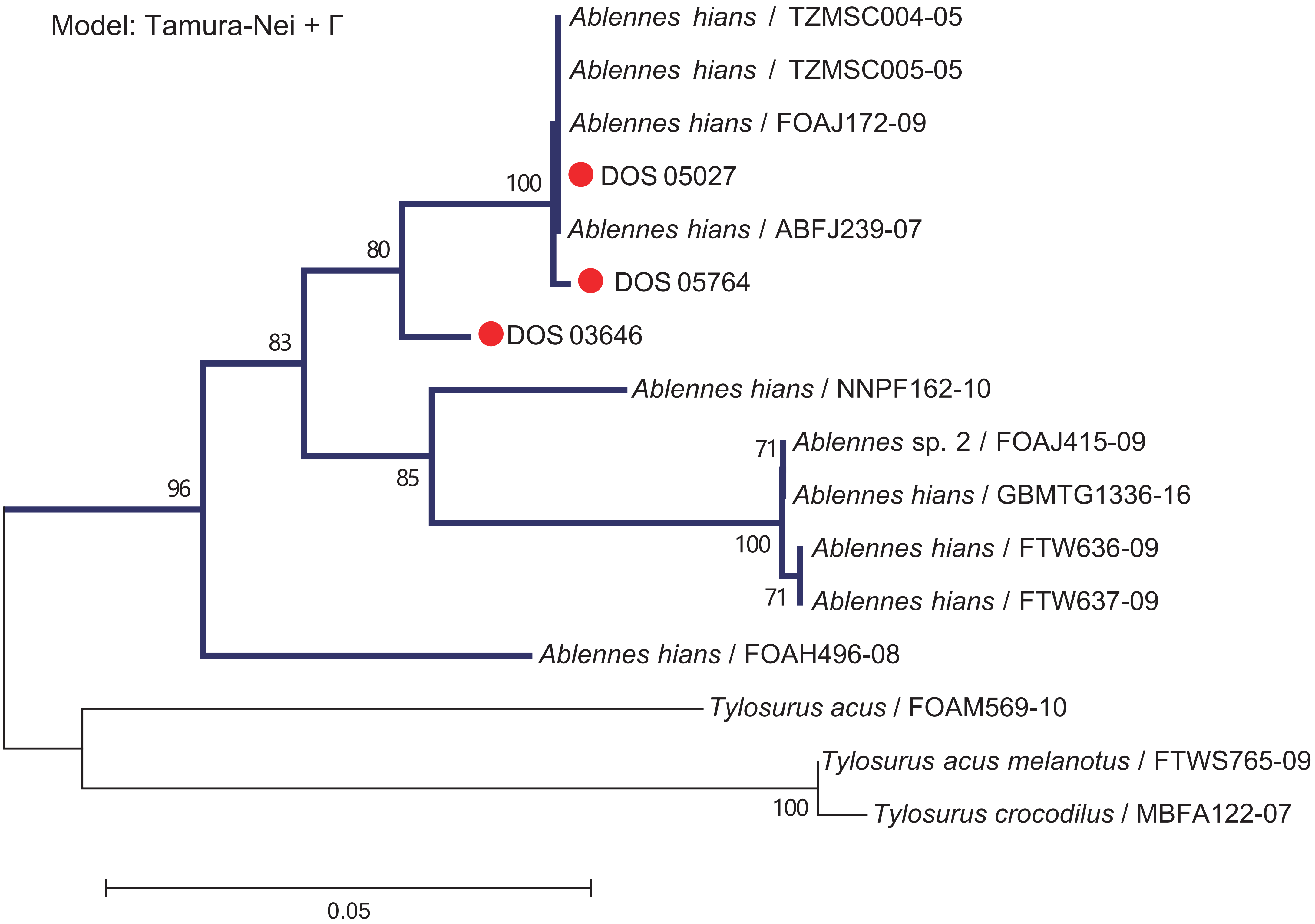

Model: Tamura-Nei + Γ
Ablennes hians / TZMSC004-05
Ablennes hians / TZMSC005-05
Ablennes hians / FOAJ172-09
DOS 05027
Ablennes hians / ABFJ239-07
DOS 05764
DOS 03646
Ablennes hians / NNPF162-10
Ablennes sp. 2 / FOAJ415-09
Ablennes hians / GBMTG1336-16
Ablennes hians / FTW636-09
Ablennes hians / FTW637-09
Ablennes hians / FOAH496-08
Tylosurus acus / FOAM569-10
Tylosurus acus melanotus / FTWS765-09
Tylosurus crocodilus / MBFA122-07
100
80
83
85
96
71
100
71
100
0.05

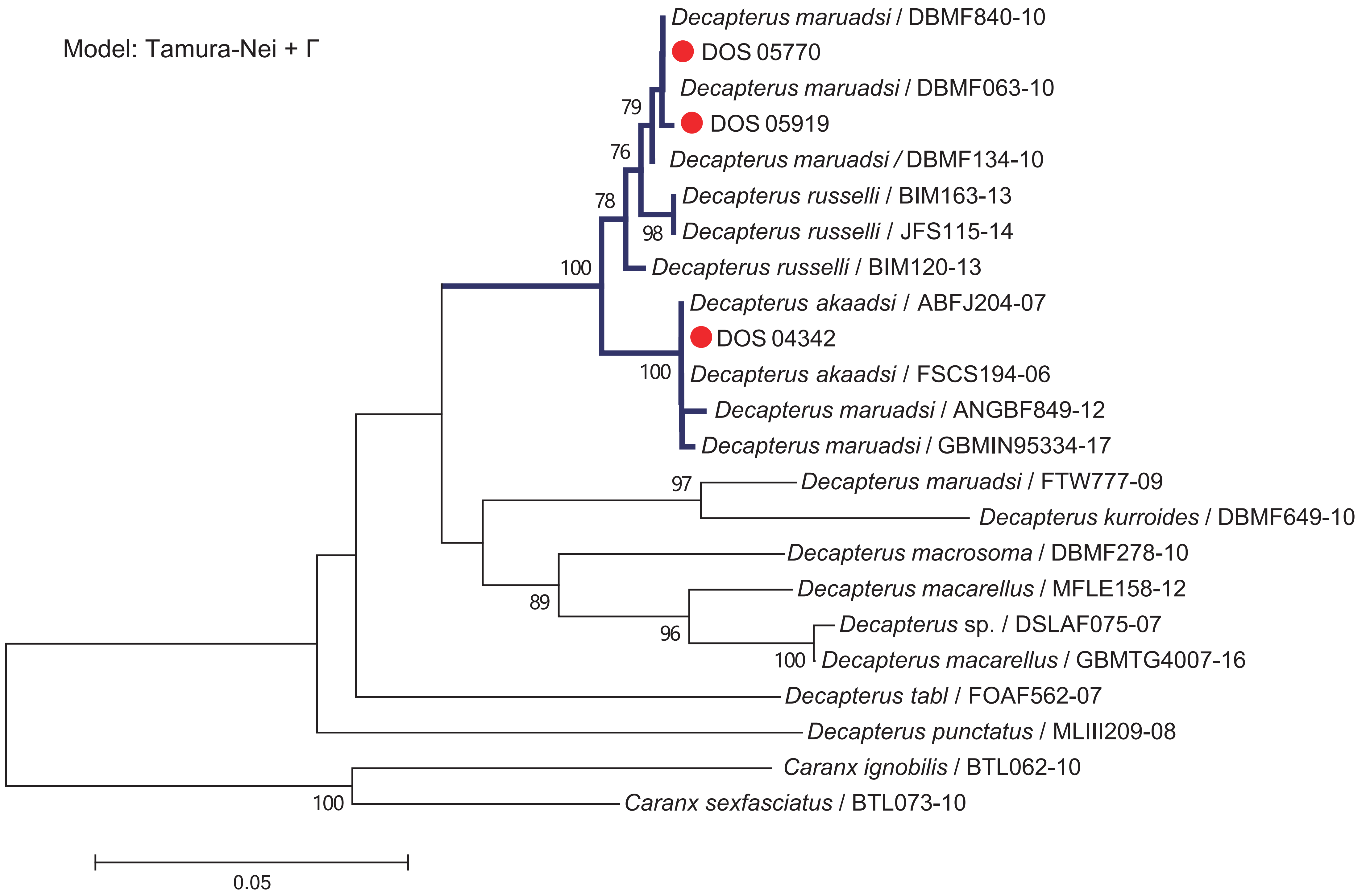
Model: Tamura-Nei + Γ
Decapterus maruadsi / DBMF840-10
DOS 05770
Decapterus maruadsi / DBMF063-10
DOS 05919
Decapterus maruadsi / DBMF134-10
Decapterus russelli / BIM163-13
Decapterus russelli / JFS115-14
Decapterus russelli / BIM120-13
Decapterus akaadsi / ABFJ204-07
DOS 04342
Decapterus akaadsi / FSCS194-06
Decapterus maruadsi / ANGBF849-12
Decapterus maruadsi / GBMIN95334-17
Decapterus maruadsi / FTW777-09
Decapterus kurroides / DBMF649-10
Decapterus macrosoma / DBMF278-10
Decapterus macarellus / MFLE158-12
Decapterus sp. / DSLAF075-07
Decapterus macarellus / GBMTG4007-16
Decapterus tabl / FOAF562-07
Decapterus punctatus / MLIII209-08
Caranx ignobilis / BTL062-10
Caranx sexfasciatus / BTL073-10
79
76
78
98
100
100
97
89
96
100
100
0.05
