# Supplementary material for: DNA barcoding of coastal ray-finned fishes in Vietnam
Source: PLoS One. 2019 Sep 19;14(9):e0222631. doi: 10.1371/journal.pone.0222631 (PMC6752846; doi:10.1371/journal.pone.0222631)
Supplement: S1 Table — NR–New recoded species in Vietnam; ND–Not yet determined; N, C, S–northern, central, and southern areas in Vietnam; *and** denote 10 species with intraspecific distances more than 2% (in Table 3); species in bold were distributed in all areas; ● species recorded in this study; ○ species recorded from Nguyen et al. [27]; numbers in parentheses represent total species excluding those recorded in Nguyen et al. [27]. (DOC) [file pone.0222631.s003.doc]

**S1 Table. The list of fish species DNA barcoded in this study.**

| Order | Family | Scientific name | NR/  ND | Distribution | | | GenBank Accession no. |
| --- | --- | --- | --- | --- | --- | --- | --- |
|  |  |  |  | N | C | S |  |
| Acanthuriformes | Acanthuridae | *Ctenochaetus binotatus* |  |  | ● |  | MK777057–MK777058 |
|  |  | *Naso brevirostris* |  |  | ● |  | MK777059–MK777061 |
|  |  | *Naso reticulatus* | NR |  | ● |  | MK777062–MK777063 |
| Albuliformes | Albulidae | *Albula glossodonta* | NR |  | ● |  | MK777064 |
| Anabantiformes | Anabantidae | *Anabas testudineus* |  | ● |  |  | MK777065 |
| Anguilliformes | Congridae | *Ariosoma meeki* |  |  | ● |  | MK777066–MK777069 |
|  |  | *Ariosoma* sp. 01 | ND |  | ● |  | MK777070 |
|  |  | *Bathymyrus simus* |  |  | ● |  | MK777071 |
|  |  | *Conger cinereus* |  |  | ● |  | MK777072–MK777073 |
|  |  | *Parabathymyrus macrophthalmus* |  |  | ● |  | MK777074 |
|  |  | *Uroconger lepturus** |  |  | ● |  | MK777075 |
|  |  | *Uroconger lepturus*** |  |  | ● |  | MK777076–MK777077 |
|  |  | *Uroconger* sp. 01 | ND |  |  | ● | MK777078 |
|  | Muraenesocidae | *Congresox talabonoides* |  |  |  | ● | MK777079 |
|  |  | *Muraenesox bagio* |  | ● |  | ● | MK777080–MK777087 |
|  | Muraenidae | *Echidna polyzona* |  |  | ● |  | MK777088 |
|  |  | *Gymnothorax emmae* |  |  | ● |  | MK777089 |
|  |  | *Gymnothorax longinquus* | NR |  |  | ● | MK777090 |
|  |  | *Gymnothorax minor* |  | ● | ● |  | MK777091–MK777092 |
|  |  | *Gymnothorax niphostigmus* | NR |  | ● |  | MF774814 |
|  |  | *Gymnothorax reevesii* |  | ● |  | ● | MK777094–MK777095 |
|  |  | *Strophidon sathete* |  |  | ● |  | MK777096 |
|  | Ophichthidae | *Ophichthus* cf. *apicalis* |  | ● |  |  | MK777097 |
|  |  | *Ophichthus* cf. *asakusae* |  |  | ● |  | MK777103–MK777104 |
|  |  | *Ophichthus lithinus* |  |  |  | ● | MK777098 |
|  |  | *Ophichthus* sp. 01 | ND |  |  | ● | MK777099–MK777101 |
|  |  | *Pisodonophis cancrivorus* |  |  | ● |  | MK777102 |
| Atheriniformes | Atherinidae | *Atherinomorus lacunosus* |  |  | ● | ● | MK777105–MK777106 |
|  |  | *Hypoatherina valenciennei* |  | ● |  |  | MK777107–MK777109 |
| Aulopiformes | Synodontidae | *Harpadon nehereus* |  |  | ● |  | MK777110 |
|  |  | *Saurida elongata* |  | ● |  |  | MK777111–MK777113 |
|  |  | *Saurida tumbil* |  | ● |  |  | MK777114 |
|  |  | *Saurida undosquamis* |  |  | ● |  | MK777115 |
|  |  | *Synodus hoshinonis* |  |  |  | ● | MK777116 |
|  |  | *Synodus* sp. 01 | ND |  | ● |  | MK777117 |
|  |  | *Synodus* sp. 02 | ND |  | ● |  | MK777118 |
|  |  | *Trachinocephalus myops* |  | ● | ● |  | MK777119–MK777121 |
| Batrachoidiformes | Batrachoididae | *Allenbatrachus grunniens* |  |  | ● |  | MK777122 |
|  |  | *Batrachomoeus* cf. *trispinosus* |  |  |  | ● | MK777123 |
| Beloniformes | Belonidae | *Ablennes hians** |  |  | ● | ● | MK777125–MK777126 |
|  |  | *Ablennes hians*** |  | ● |  |  | MK777127 |
|  |  | *Cololabis saira* | NR | ● |  |  | MK777128 |
|  |  | *Strongylura incisa* | NR |  |  | ● | MK777129 |
|  |  | *Strongylura leiura* |  |  | ● |  | MK777130 |
|  |  | *Strongylura strongylura* |  | ● |  |  | MK777131 |
|  |  | *Tylosurus crocodilus* |  |  | ● |  | MK777132 |
|  | Adrianichthyidae | *Oryzias haugiangensis* |  |  | ● |  | MK777124 |
|  | Exocoetidae | *Cypselurus* sp. 01 | ND |  | ● |  | MK777133 |
|  |  | *Parexocoetus brachypterus* |  |  | ● |  | MK777134 |
|  |  | *Parexocoetus mento* |  |  | ● |  | MK777135–MK777137 |
|  | Hemiramphidae | *Hemiramphus* cf. *archipelagicus* |  |  | ● |  | MK777139 |
|  |  | *Hemiramphus far* |  |  | ● |  | MK777138 |
|  |  | *Hyporhamphus limbatus* |  | ● |  |  | MK777140 |
|  |  | *Hyporhamphus quoyi* |  |  |  | ● | MK777141 |
|  |  | *Zenarchopterus* sp. 01 | ND |  | ● |  | MK777142 |
| Beryciformes | Berycidae | *Centroberyx druzhinini* | NR |  | ● |  | MK777143 |
| Blenniiformes | Blenniidae | *Petroscirtes variabilis* |  |  | ● |  | MK777144 |
| Carangiformes | Carangidae | *Alectis ciliaris* |  | ● |  |  | MK777145 |
|  |  | *Alectis indica* |  | ● |  |  | MK777146 |
|  |  | *Alepes djedaba* |  |  |  | ● | MK777147 |
|  |  | *Alepes kleinii* |  | ● | ● |  | MK777148–MK777151 |
|  |  | *Alepes melanoptera* |  | ● |  | ● | MK777152–MK777154 |
|  |  | *Atropus atropos* |  |  |  | ● | MK777155 |
|  |  | *Atule mate* |  |  | ● | ● | MK777156–MK777160 |
|  |  | *Carangoides coeruleopinnatus* |  |  | ● | ● | MK777161–MK777163 |
|  |  | *Carangoides equula* |  |  | ● |  | MK777164 |
|  |  | *Carangoides ferdau* |  | ● |  |  | MK777165 |
|  |  | *Carangoides hedlandensis* |  |  | ● |  | MK777166 |
|  |  | *Carangoides malabaricus* |  | ● | ● |  | MK777167–MK777170 |
|  |  | *Carangoides praeustus* |  |  | ● |  | MK777171 |
|  |  | *Decapterus maruadsi** |  |  | ● |  | MK777172 |
|  |  | *Decapterus maruadsi*** |  |  |  | ● | MK777173–MK777174 |
|  |  | *Megalaspis cordyla* |  | ● |  | ● | MK777175–MK777177 |
|  |  | *Parastromateus niger* |  | ● |  | ● | MK777178–MK777180 |
|  |  | *Scomberoides commersonnianus* |  |  | ● |  | MK777181 |
|  |  | *Scomberoides lysan* |  |  | ● |  | MK777182 |
|  |  | *Scomberoides tol* |  |  |  | ● | MK777183 |
|  |  | *Selar crumenophthalmus* |  |  |  | ● | MK777184 |
|  |  | *Selaroides leptolepis* |  |  | ● |  | MK777185 |
|  |  | *Seriola quinqueradiata* | NR | ● |  |  | MK777186 |
|  |  | *Seriolina nigrofasciata** |  |  | ● | ● | MK777187–MK777188 |
|  |  | *Seriolina nigrofasciata*** |  |  | ● |  | MK777189 |
|  |  | *Trachinotus baillonii* |  |  | ● |  | MK777190–MK777191 |
|  |  | *Trachinotus ovatus* |  | ● |  |  | MK777192 |
|  |  | *Trachurus japonicus* |  | ● |  |  | MK777193 |
|  |  | *Uraspis uraspis* |  |  | ● | ● | MK777194–MK777197 |
|  | Coryphaenidae | *Coryphaena hippurus* |  |  | ● | ● | MK777198–MK777201 |
|  | Echeneidae | *Echeneis naucrates* |  |  | ● |  | MK777202 |
|  | Rachycentridae | *Rachycentron canadum* |  | ● |  | ● | MK777203–MK777204 |
| Centrarchiformes | Kyphosidae | *Kyphosus vaigiensis* |  | ● | ● |  | MK777205–MK777206 |
|  | Terapontidae | *Pelates quadrilineatus* |  |  | ● |  | MK777207 |
|  |  | *Terapon jarbua* |  |  | ● | ● | MK777208–MK777209 |
|  |  | ***Terapon theraps*** |  | ● | ● | ● | MK777210–MK777213 |
| Chaetodontiformes | Chaetodontidae | *Chaetodon auriga* |  |  | ● |  | MK777214–MK777215 |
|  |  | *Chaetodon guentheri* |  |  | ● |  | MK777216 |
|  |  | *Chaetodon modestus* | NR |  | ● |  | MK777217 |
|  |  | *Chaetodon ornatissimus* |  |  | ● |  | MK777218 |
|  |  | *Chelmon rostratus* |  | ● |  |  | MK777219 |
|  |  | *Heniochus chrysostomus* |  |  | ● |  | MK777220 |
|  |  | *Parachaetodon ocellatus* |  |  |  | ● | MK777221 |
|  | Leiognathidae | *Eubleekeria jonesi* |  |  | ● |  | MK777222–MK777223 |
|  |  | *Gazza minuta* |  |  | ● |  | MK777224 |
|  |  | *Karalla daura* |  |  | ● |  | MK777225 |
|  |  | *Leiognathus berbis* | NR | ● | ● |  | MK777226–MK777230 |
|  |  | *Leiognathus brevirostris* |  |  | ● | ● | MK777231–MK777232 |
|  |  | *Leiognathus equulus* |  | ● | ● |  | MK777233–MK777234 |
|  |  | *Nuchequula gerreoides* |  | ● |  |  | MK777235 |
|  |  | *Photolateralis stercorarius* |  |  | ● | ● | MK777236–MK777238 |
|  |  | *Secutor insidiator* |  | ● |  |  | MK777239 |
|  |  | *Secutor megalolepis* |  | ● | ● |  | MK777240–MK777243 |
| Clupeiformes | Clupeidae | *Anodontostoma chacunda* |  |  |  | ● | MK777247–MK777248 |
|  |  | *Corica laciniata* |  |  |  | ● | MK777249 |
|  |  | *Dussumieria elopsoides* |  | ● |  |  | MK777250–MK777251 |
|  |  | *Escualosa thoracata* |  | ● |  | ● | MK777252–MK777256 |
|  |  | *Sardinella gibbosa* |  |  | ● |  | MK777257 |
|  |  | *Sardinella melanura* |  | ● | ● |  | MK777258–MK777259 |
|  | Chirocentridae | *Chirocentrus dorab* |  | ● |  | ● | MK777245–MK777246 |
|  | Engraulidae | *Coilia mystus* |  | ● |  |  | MK777260–MK777261 |
|  |  | *Encrasicholina heteroloba* |  | ● | ● |  | MK777262–MK777263 |
|  |  | *Setipinna melanochir* |  |  |  | ● | MK777264 |
|  |  | *Stolephorus commersonnii* |  | ● |  |  | MK777265–MK777266 |
|  |  | *Stolephorus waitei* |  |  |  | ● | MK777267–MK777268 |
|  |  | *Thryssa dussumieri* |  | ● |  |  | MK777269 |
|  |  | *Thryssa hamiltonii* |  | ● |  |  | MK777270 |
|  |  | *Thryssa setirostris* |  | ● |  |  | MK777271 |
|  | Pristigasteridae | *Ilisha melastoma* |  | ● |  |  | MK777272–MK777273 |
| Elopiformes | Elopidae | *Elops machnata* |  |  |  | ● | MK777275 |
|  | Megalopidae | *Megalops cyprinoides* |  |  | ● |  | MK777276 |
| Ephippiformes | Drepaneidae | ***Drepane punctata*** |  | ● | ● | ● | MK777277–MK777281 |
|  | Ephippidae | *Platax teira* |  | ● | ● |  | MK777282–MK777284 |
| Gadiformes | Bregmacerotidae | *Bregmaceros* sp. 01 | ND | ● |  |  | MK777285 |
| Gerreiformes | Gerreidae | *Gerres erythrourus* |  |  |  | ● | MK777286 |
|  |  | *Gerres filamentosus* |  | ● |  |  | MK777287 |
|  |  | *Gerres limbatus* |  | ● |  | ● | MK777288–MK777289 |
|  |  | *Gerres oyena* |  |  | ● | ● | MK777290–MK777291 |
|  |  | *Gerres shima* |  |  | ● |  | MK777292–MK777293 |
| Gobiiformes | Gobiidae | *Acentrogobius caninus* |  | ● |  |  | MK777311 |
|  |  | *Acentrogobius chlorostigmatoides* |  |  |  | ○ | MK777312 |
|  |  | *Acentrogobius nebulosus* |  |  | ○ |  | MK777313 |
|  |  | *Acentrogobius* sp. 01 | ND |  | ● |  | MK777314 |
|  |  | *Acentrogobius viridipunctatus* |  |  |  | ○ | MK777315 |
|  |  | *Amblyeleotris macronema* | NR |  |  | ● | MK777316 |
|  |  | *Amblygobius phalaena* |  | ○ |  |  | MK777317 |
|  |  | *Bathygobius fuscus* |  |  | ○ |  | MK777318 |
|  |  | *Cryptocentrus cebuanus* | NR |  |  | ● | MK777320–MK777322 |
|  |  | *Cryptocentrus leptocephalus* |  |  | ○ |  | MK777319 |
|  |  | *Favonigobius reichei* |  |  | ○ |  | MK777323 |
|  |  | *Glossogobius aureus* |  |  | ○ |  | MK777324 |
|  |  | *Glossogobius bicirrhosus* |  |  | ● |  | MK777325–MK777326 |
|  |  | *Glossogobius giuris* |  |  | ● | ○ | MK777327–MK777328 |
|  |  | *Mahidolia mystacina* |  |  |  | ● | MK777329–MK777330 |
|  |  | *Oplopomus caninoides* |  |  |  | ● | MK777331 |
|  |  | *Parachaeturichthys polynema* |  | ● |  |  | MK777332–MK777333 |
|  |  | *Psammogobius biocellatus* |  | ○ | ● |  | MK777334–MK777337 |
|  |  | *Valenciennea puellaris* |  |  | ○ |  | MK777338 |
|  | Eleotridae | *Bostrychus* cf. *sinensis* |  |  |  | ● | MK777294 |
|  |  | *Butis amboinensis* |  |  | ● |  | MK777295 |
|  |  | *Butis koilomatodon* |  |  |  | ● | MK777296 |
|  |  | *Eleotris acanthopoma* |  |  | ● |  | MK777297 |
|  |  | *Eleotris fusca* |  |  |  | ● | MK777298–MK777308 |
|  |  | *Eleotris melanosoma* |  |  | ● |  | MK777309 |
|  |  | *Ophiocara porocephala* | NR |  |  | ● | MK777310 |
|  | Oxudercidae | *Amblyotrypauchen arctocephalus* |  |  | ○ |  | MK777339 |
|  |  | *Apocryptodon madurensis* |  |  |  | ● | MK777354 |
|  |  | *Boleophthalmus boddarti* |  |  |  | ○ | MK777340 |
|  |  | *Hemigobius melanurus* | NR |  |  | ● | MK777341 |
|  |  | *Luciogobius guttatus* | NR | ● |  |  | MK777342 |
|  |  | *Oligolepis acutipennis* |  |  | ○ |  | MK777343 |
|  |  | *Oligolepis* sp. 01 | ND |  |  | ○ | MK777344 |
|  |  | ***Oxyurichthys auchenolepis*** |  | ○ | ○ | ● | MK777346–MK777349 |
|  |  | *Oxyurichthys longicauda* |  | ○ |  |  | MK777351 |
|  |  | *Oxyurichthys microlepis* |  |  | ● |  | MK777350 |
|  |  | *Oxyurichthys* sp. 01 | ND |  | ○ |  | MK777352 |
|  |  | *Parapocryptes serperaster* |  |  |  | ○ | MK777353 |
|  |  | *Periophthalmus* sp. 01 | ND |  |  | ● | MK777355 |
|  |  | *Pseudapocryptes elongatus* |  |  |  | ○ | MK777356 |
|  |  | *Pseudogobius javanicus* |  |  | ○ | ● | MK777357–MK777358 |
|  |  | *Stenogobius mekongensis* |  |  |  | ○ | MK777345 |
|  |  | *Stigmatogobius pleurostigma* |  |  |  | ○ | MK777359 |
|  |  | *Tridentiger bifasciatus* |  | ● |  |  | MK777360–MK777362 |
|  |  | *Trypauchen vagina** |  | ● |  |  | MK777363–MK777364 |
|  |  | *Trypauchen vagina*** |  |  |  | ○ | MK777365 |
| Gonorynchiformes | Chanidae | *Chanos chanos* |  |  | ● |  | MK777366 |
|  | Gonorynchidae | *Gonorynchus abbreviatus* |  |  | ● |  | MK777367 |
| Holocentriformes | Holocentridae | *Myripristis botche* |  |  | ● |  | MK777368 |
|  |  | *Ostichthys japonicus* |  |  | ● |  | MK777369 |
|  |  | ***Sargocentron rubrum*** |  | ● | ● | ● | MK777370–MK777374 |
| Istiophoriformes | Istiophoridae | *Istiompax indica* |  |  | ● |  | MK777375 |
|  |  | *Istiophorus platypterus* |  |  |  | ● | MK777376 |
| Kurtiformes | Apogonidae | *Apogon* sp. 01 | ND |  | ● | ● | MK777377–MK777379 |
|  |  | *Apogonichthyoides cathetogramma* | NR |  | ● |  | MK777380 |
|  |  | *Archamia bleekeri* |  |  | ● |  | MK777381 |
|  |  | *Jaydia striata* |  |  | ● |  | MK777382 |
|  |  | ***Jaydia truncata*** |  | ● | ● | ● | MK777383–MK777387 |
|  |  | *Jaydia striatodes* |  | ● |  |  | MK777388 |
|  |  | *Ostorhinchus cheni* | NR |  | ● |  | MK777389 |
|  |  | *Ostorhinchus fleurieu* |  |  | ● |  | MK777390 |
|  |  | *Ostorhinchus novemfasciatus* | NR |  | ● |  | MK777391 |
|  |  | ***Ostorhinchus pleuron*** |  | ● | ● | ● | MK777392–MK777394 |
|  |  | *Ostorhinchus semilineatus* |  |  | ● |  | MK777395 |
|  |  | *Siphamia* sp. 01 | ND |  | ● |  | MK777846 |
|  |  | *Taeniamia fucata* |  |  | ● |  | MK777396 |
|  |  | *Taeniamia* sp. 01 | ND |  |  | ● | MK777397 |
| Labriformes | Labridae | *Cheilinus chlorourus* |  |  | ● |  | MK777398–MK777399 |
|  |  | *Cheilinus trilobatus* |  |  | ● |  | MK777400 |
|  |  | *Halichoeres bicolor* |  |  | ● | ● | MK777401–MK777404 |
|  |  | *Halichoeres nigrescens* |  |  | ● |  | MK777405 |
|  |  | *Iniistius verrens* | NR |  | ● | ● | MK777406–MK777408 |
|  |  | *Iniistius evides* |  |  |  | ● | MK777409 |
|  |  | *Leptojulis lambdastigma* | NR |  | ● |  | MK777410–MK777411 |
|  |  | *Stethojulis terina* |  |  | ● |  | MK777412 |
|  | Scaridae | *Scarus rivulatus* |  |  | ● |  | MK777413 |
| Lobotiformes | Lobotidae | *Datnioides polota* |  |  |  | ● | MK777414 |
|  |  | *Lobotes surinamensis* |  |  |  | ● | MK777415 |
| Lophiiformes | Antennariidae | *Antennarius striatus* |  |  | ● |  | MK777416 |
|  | Lophiidae | *Lophius litulon* |  |  | ● | ● | MK777417–MK777418 |
| Lutjaniformes | Lutjanidae | *Caesio caerulaurea* |  |  |  | ● | MK777432 |
|  |  | *Caesio cuning* |  | ● | ● |  | MK777433–MK777434 |
|  |  | *Dipterygonotus balteatus* |  |  | ● | ● | MK777435–MK777436 |
|  |  | *Lutjanus argentimaculatus* |  | ● | ● |  | MK777437–MK777439 |
|  |  | *Lutjanus carponotatus* |  |  |  | ● | MK777440 |
|  |  | *Lutjanus erythropterus* |  | ● |  | ● | MK777441–MK777443 |
|  |  | *Lutjanus fulviflamma* |  |  | ● |  | MK777444–MK777446 |
|  |  | *Lutjanus johnii* |  | ● |  | ● | MK777447–MK777449 |
|  |  | *Lutjanus lutjanus* |  |  | ● | ● | MK777450–MK777452 |
|  |  | *Lutjanus madras* |  |  |  | ● | MK777453 |
|  |  | *Lutjanus monostigma* |  | ● |  | ● | MK777454–MK777456 |
|  |  | *Lutjanus quinquelineatus* |  |  |  | ● | MK777457–MK777458 |
|  |  | *Lutjanus russellii* |  |  | ● | ● | MK777459–MK777460 |
|  |  | *Lutjanus vitta* |  | ● |  | ● | MK777461–MK777462 |
|  |  | *Pristipomoides multidens* |  |  | ● |  | MK777463 |
|  |  | *Pterocaesio chrysozona* |  |  |  | ● | MK777464 |
|  |  | *Pterocaesio digramma* |  |  | ● |  | MK777465 |
|  |  | *Symphorus nematophorus* |  |  | ● |  | MK777466 |
|  | Haemulidae | ***Diagramma pictum*** |  | ● | ● | ● | MK777419–MK777424 |
|  |  | *Plectorhinchus gibbosus* |  |  | ● |  | MK777425–MK777426 |
|  |  | *Pomadasys kaakan* |  | ● |  |  | MK777427 |
|  |  | *Pomadasys maculatus* |  | ● | ● |  | MK777428–MK777431 |
| Mugiliformes | Mugilidae | *Chelon affinis* |  | ● |  |  | MK777477–MK777478 |
|  |  | *Chelon subviridis* |  |  | ● |  | MK777467 |
|  |  | *Moolgarda cunnesius* |  |  | ● |  | MK777468 |
|  |  | *Moolgarda perusii* |  | ● | ● |  | MK777469–MK777472 |
|  |  | *Mugil cephalus* |  |  | ● |  | MK777473–MK777474 |
|  |  | *Paramugil parmatus* |  |  |  | ● | MK777475–MK777476 |
| Ophidiiformes | Ophidiidae | *Brotula townsendi* | NR |  | ● |  | MK777479 |
|  |  | *Ophidion muraenolepis* |  |  | ● |  | MK777480 |
| Order-level incertae sedis in Carangaria | Polynemidae | *Eleutheronema rhadinum* |  | ● |  |  | MK777485–MK777486 |
|  |  | *Eleutheronema tetradactylum* |  |  |  | ● | MK777487 |
|  |  | *Polynemus melanochir* |  |  |  | ● | MK777488 |
|  | Sphyraenidae | *Sphyraena barracuda* |  |  | ● |  | MK777489–MK777490 |
|  |  | *Sphyraena forsteri* |  |  | ● |  | MK777495 |
|  |  | *Sphyraena jello* |  | ● |  |  | MK777491 |
|  |  | *Sphyraena pinguis* |  |  |  | ● | MK777492 |
|  |  | *Sphyraena putnamae* |  | ● |  | ● | MK777493–MK777494 |
|  |  | *Sphyraena* sp. 01 | ND |  |  | ● | MK777496 |
|  | Centropomidae | *Lates calcarifer* |  | ● |  |  | MK777481 |
|  |  | *Psammoperca waigiensis* |  |  | ● |  | MK777482 |
|  | Lactariidae | *Lactarius lactarius* |  |  | ● |  | MK777483 |
|  | Menidae | *Mene maculata* |  |  |  | ● | MK777484 |
| Order-level incertae sedis in Eupercaria | Pomacanthidae | *Apolemichthys trimaculatus* |  |  | ● |  | MK777503 |
|  |  | *Chaetodontoplus septentrionalis* |  |  | ● |  | MK777504 |
|  |  | *Pomacanthus semicirculatus* |  |  | ● |  | MK777505 |
|  | Emmelichthyidae | *Erythrocles scintillans* | NR |  | ● |  | MK777497 |
|  | Malacanthidae | *Branchiostegus albus* |  | ● |  |  | MK777498–MK777499 |
|  |  | *Branchiostegus argentatus* |  |  | ● | ● | MK777500–MK777501 |
|  | Monodactylidae | *Monodactylus argenteus* |  |  | ● |  | MK777502 |
|  | Scatophagidae | *Scatophagus argus** |  | ● |  |  | MK777506 |
|  |  | *Scatophagus argus*** |  |  | ● | ● | MK777507–MK777508 |
|  | Sciaenidae | *Dendrophysa russelii* |  | ● |  | ● | MK777509–MK777511 |
|  |  | *Johnius amblycephalus* |  |  | ● |  | MK777512–MK777513 |
|  |  | *Johnius belangerii* |  | ● |  |  | MK777514–MK777515 |
|  |  | *Nibea soldado* |  |  |  | ● | MK777516 |
|  |  | *Otolithes ruber* |  |  |  | ● | MK777517 |
|  |  | *Pennahia anea* |  | ● |  |  | MK777518–MK777520 |
|  |  | *Pennahia pawak* |  | ● |  |  | MK777521 |
|  |  | *Sciaenops ocellatus* | NR | ● |  |  | MK777522 |
|  | Siganidae | ***Siganus canaliculatus*** |  | ● | ● | ● | MK777523–MK777526 |
|  |  | *Siganus guttatus* |  |  | ● |  | MK777527 |
|  |  | *Siganus javus* |  |  |  | ● | MK777528 |
|  |  | *Siganus punctatus* |  |  |  | ● | MK777529 |
|  |  | *Siganus virgatus* |  |  |  | ● | MK777530 |
|  | Sillaginidae | ***Sillago aeolus*** |  | ● | ● | ● | MK777531–MK777533 |
|  |  | *Sillago asiatica* |  |  | ● | ● | MK777534–MK777536 |
|  |  | *Sillago ingenuua* |  |  |  | ● | MK777537 |
|  |  | *Sillago lutea* | NR |  | ● |  | MK777540 |
|  |  | *Sillago sihama* |  | ● |  | ● | MK777538–MK777539 |
| Order-level incertae sedis in Ovalentaria | Pomacentridae | *Abudefduf bengalensis* |  | ● |  | ● | MK777548–MK777550 |
|  |  | *Chromis mirationis* | NR |  | ● |  | MK777551 |
|  |  | *Pomacentrus geminospilus* | NR |  |  | ● | MK777552 |
|  |  | *Pristotis obtusirostris* |  |  | ● |  | MK777558–MK777559 |
|  |  | *Stegastes fasciolatus* |  |  | ● |  | MK777553 |
|  |  | *Teixeirichthys jordani* | NR |  | ● |  | MK777554–MK777557 |
|  | Ambassidae | *Ambassis dussumieri* | NR |  | ● |  | MK777541 |
|  |  | *Ambassis interrupta* |  |  | ● |  | MK777542 |
|  |  | *Ambassis marianus* | NR |  |  | ● | MK777543 |
|  |  | *Ambassis nalua* |  |  | ● |  | MK777544 |
|  |  | *Ambassis urotaenia* |  |  | ● |  | MK777545 |
|  |  | *Parambassis wolffii* |  |  |  | ● | MK777546 |
|  | Opistognathidae | *Opistognathus* sp. 01 | ND |  | ● |  | MK777547 |
|  | Pseudochromidae | *Congrogadus subducens* |  |  |  | ● | MK777560 |
| Pempheriformes | Champsodontidae | *Champsodon vorax* |  |  | ● |  | MK777561 |
|  | Pempheridae | *Parapriacanthus ransonneti* |  |  | ● |  | MK777562 |
|  |  | *Pempheris schwenkii* |  |  | ● |  | MK777563 |
|  | Pentacerotidae | *Histiopterus typus* |  |  | ● |  | MK777564 |
| Perciformes | Scorpaenidae | *Apistus carinatus* |  |  | ● |  | MK777582 |
|  |  | *Erosa erosa* |  |  | ● |  | MK777583 |
|  |  | *Inimicus sinensis* |  |  | ● |  | MK777584–MK777585 |
|  |  | *Minous coccineus* | NR |  | ● |  | MK777586 |
|  |  | *Minous monodactylus* |  | ● |  |  | MK777587 |
|  |  | *Minous quincarinatus* | NR |  | ● |  | MK777588 |
|  |  | *Parapterois heterura* |  |  | ● |  | MK777589 |
|  |  | *Pterois russelii* |  |  |  | ● | MK777590 |
|  |  | *Scorpaenopsis barbata* | NR | ● |  |  | MK777591 |
|  |  | *Scorpaenopsis neglecta* |  |  | ● |  | MK777592–MK777593 |
|  |  | *Sebastiscus marmoratus* |  | ● |  |  | MK777594 |
|  | Aploactinidae | *Erisphex simplex* | NR |  | ● |  | MK777565 |
|  | Peristediidae | *Satyrichthys rieffeli* |  |  | ● |  | MK777566 |
|  | Platycephalidae | *Inegocia japonica* |  | ● | ● |  | MK777567–MK777571 |
|  |  | *Kumococius rodericensis* |  |  |  | ● | MK777572 |
|  |  | *Kumococius* sp. 01 | ND |  |  | ● | MK777573 |
|  |  | *Platycephalus indicus** |  | ● |  |  | MK777574 |
|  |  | *Platycephalus indicus*** |  | ● |  |  | MK777575 |
|  |  | *Ratabulus* sp. 01 | ND |  |  | ● | MK777576 |
|  |  | *Rogadius asper* |  |  | ● |  | MK777578 |
|  |  | *Rogadius* sp. 01 | ND |  |  | ● | MK777579–MK777580 |
|  |  | *Sunagocia carbunculus* |  |  |  | ● | MK777577 |
|  |  | *Thysanophrys celebica* |  |  | ● |  | MK777581 |
|  | Serranidae | *Cephalopholis boenak* |  | ● |  | ● | MK777595–MK777597 |
|  |  | *Chelidoperca pleurospilus* | NR |  | ● |  | MK777598 |
|  |  | *Chelidoperca santosi* | NR |  | ● |  | MK777599 |
|  |  | *Diploprion bifasciatum* |  | ● |  | ● | MK777600–MK777601 |
|  |  | *Epinephelus amblycephalus* |  |  | ● |  | MK777602 |
|  |  | ***Epinephelus areolatus*** |  | ● | ● | ● | MK777603–MK777608 |
|  |  | *Epinephelus awoara* |  |  | ● |  | MK777609 |
|  |  | *Epinephelus bleekeri* |  | ● |  |  | MK777610–MK777611 |
|  |  | *Epinephelus coioides* |  | ● |  | ● | MK777612–MK777615 |
|  |  | *Epinephelus epistictus* |  |  | ● |  | MK777616 |
|  |  | *Epinephelus fasciatus* |  |  | ● |  | MK777617 |
|  |  | *Epinephelus fuscoguttatus* |  | ● |  |  | MK777618 |
|  |  | *Epinephelus latifasciatus* |  |  |  |  | MK777619 |
|  |  | *Epinephelus maculatus* |  |  | ● |  | MK777620 |
|  |  | *Epinephelus quoyanus* |  |  |  | ● | MK777621 |
|  |  | ***Epinephelus sexfasciatus*** |  | ● | ● | ● | MK777622–MK777626 |
|  |  | *Epinephelus stictus* |  |  |  | ● | MK777627 |
|  |  | *Grammistes sexlineatus* |  |  | ● |  | MK777628 |
|  |  | *Plectropomus leopardus* |  |  |  | ● | MK777629 |
|  |  | *Triso dermopterus* |  |  | ● |  | MK777630 |
|  | Synanceiidae | *Trachicephalus uranoscopus* |  | ● | ● |  | MK777631–MK777632 |
|  | Tetrarogidae | *Tetraroge barbata* | NR |  | ● |  | MK777633 |
|  | Triglidae | *Lepidotrigla abyssalis* |  |  | ● |  | MK777634 |
|  |  | *Lepidotrigla microptera* |  | ● |  |  | MK777635 |
| Pleuronectiformes | Bothidae | *Arnoglossus polyspilus* | NR |  | ● |  | MK777636 |
|  |  | *Arnoglossus tapeinosoma* |  |  | ● |  | MK777638 |
|  |  | Bothidae sp. 01 | ND | ● |  |  | MK777637 |
|  |  | *Crossorhombus azureus* |  |  | ● |  | MK777639–MK777641 |
|  |  | *Laeops lanceolata* |  |  | ● |  | MK777642 |
|  | Cynoglossidae | *Cynoglossus arel* |  |  |  | ● | MK777643–MK777644 |
|  |  | *Cynoglossus bilineatus* |  | ● |  |  | MK777645 |
|  |  | *Cynoglossus kopsii* |  |  | ● |  | MK777646–MK777647 |
|  |  | *Cynoglossus macrolepidotus* |  | ● |  |  | MK777648–MK777650 |
|  |  | *Cynoglossus nigropinnatus* | NR |  |  | ● | MK777651 |
|  |  | *Cynoglossus oligolepis* |  |  | ● | ● | MK777652–MK777653 |
|  |  | *Cynoglossus puncticeps* |  | ● |  |  | MK777654 |
|  |  | *Cynoglossus* sp. 01 | ND |  | ● |  | MK777655–MK777657 |
|  |  | *Cynoglossus* sp. 02 | ND |  |  | ● | MK777658 |
|  |  | *Cynoglossus* sp. 03 | ND |  |  | ● | MK777659 |
|  | Paralichthyidae | *Pseudorhombus arsius* |  |  | ● |  | MK777660 |
|  |  | *Pseudorhombus javanicus* |  |  | ● | ● | MK777661–MK777662 |
|  |  | *Pseudorhombus oligodon* |  | ● |  |  | MK777663 |
|  |  | *Pseudorhombus* sp. 01 | ND |  | ● |  | MK777664 |
|  | Psettodidae | *Psettodes erumei* |  |  |  | ● | MK777665 |
|  | Samaridae | *Samaris cristatus* |  |  | ● |  | MK777666 |
|  | Soleidae | *Brachirus orientalis* |  | ● |  |  | MK777667 |
|  |  | *Dagetichthys* sp. 01 | ND |  |  | ● | MK777672 |
|  |  | *Pardachirus pavoninus* |  |  | ● | ● | MK777668–MK777670 |
|  |  | *Solea ovata* |  | ● |  |  | MK777671 |
|  |  | *Zebrias crossolepis* |  |  | ● |  | MK777673 |
|  |  | *Zebrias quagga* |  | ● | ● |  | MK777674–MK777675 |
|  |  | *Zebrias* sp. 01 | ND |  | ● |  | MK777676 |
|  |  | *Zebrias zebrinus* |  | ● |  |  | MK777677 |
| Priacanthiformes | Cepolidae | *Acanthocepola limbata* |  |  | ● |  | MK777678–MK777679 |
|  |  | *Acanthocepola krusensternii* |  |  | ● |  | MK777680 |
|  |  | *Acanthocepola* sp. 01 | ND | ● |  | ● | MK777681–MK777682 |
|  | Priacanthidae | *Priacanthus macracanthus* |  |  | ● |  | MK777683 |
|  |  | *Priacanthus tayenus* |  | ● |  | ● | MK777684–MK777688 |
|  |  | *Pristigenys niphonia* |  |  | ● |  | MK777689–MK777691 |
| Scombriformes | Ariommatidae | *Ariomma indicum* |  |  | ● |  | MK777692–MK777693 |
|  | Bramidae | *Brama orcini* | NR |  | ● |  | MK777694 |
|  | Nomeidae | *Cubiceps whiteleggii* | NR |  | ● |  | MK777695–MK777696 |
|  | Scombridae | *Auxis thazard* |  |  | ● | ● | MK777697–MK777698 |
|  |  | *Euthynnus affinis* |  |  |  | ● | MK777699 |
|  |  | ***Rastrelliger kanagurta*** |  | ● | ● | ● | MK777700–MK777703 |
|  |  | *Scomber australasicus* |  | ● |  | ● | MK777704–MK777705 |
|  |  | ***Scomberomorus commerson*** |  | ● | ● | ● | MK777706–MK777711 |
|  |  | *Scomberomorus guttatus* |  |  |  | ● | MK777712 |
|  | Trichiuridae | *Lepturacanthus savala* |  | ● |  |  | MK777713–MK777714 |
|  |  | *Trichiurus nanhaiensis* | NR |  | ● |  | MK777715 |
|  |  | *Trichiurus brevis* | NR |  |  | ● | MK777716 |
| Siluriformes | Ariidae | *Arius subrostratus* | NR |  |  | ● | MK777717 |
|  |  | *Hexanematichthys sagor* |  |  |  | ● | MK777718 |
|  |  | *Plicofollis polystaphylodon* | NR | ● |  |  | MK777719 |
|  | Plotosidae | *Plotosus lineatus** |  | ● | ● |  | MK777724–MK777725 |
|  |  | *Plotosus lineatus*** |  |  |  | ● | MK777726 |
| Spariformes | Sparidae | *Acanthopagrus latus* |  | ● |  |  | MK777773–MK777774 |
|  |  | *Acanthopagrus schlegelii* |  | ● | ● |  | MK777775–MK777776 |
|  |  | *Evynnis cardinalis* |  | ● |  |  | MK777777 |
|  |  | *Evynnis tumifrons* |  |  | ● |  | MK777778–MK777780 |
|  | Lethrinidae | *Lethrinus atkinsoni* |  |  | ● |  | MK777727–MK777729 |
|  |  | *Lethrinus genivittatus* |  |  | ● |  | MK777730 |
|  |  | *Lethrinus harak* |  |  | ● |  | MK777731 |
|  |  | *Lethrinus lentjan* |  |  | ● | ● | MK777732–MK777735 |
|  |  | *Lethrinus nebulosus* |  | ● | ● |  | MK777736–MK777739 |
|  |  | *Lethrinus ornatus* |  |  | ● |  | MK777740–MK777741 |
|  | Nemipteridae | *Nemipterus balinensoides* | NR |  |  | ● | MK777742 |
|  |  | *Nemipterus bathybius* |  |  | ● |  | MK777743 |
|  |  | *Nemipterus furcosus* |  |  | ● |  | MK777744 |
|  |  | *Nemipterus japonicus* |  | ● |  |  | MK777745–MK777748 |
|  |  | *Nemipterus marginatus* |  | ● |  |  | MK777749 |
|  |  | *Nemipterus nemurus* |  |  |  | ● | MK777750 |
|  |  | *Nemipterus virgatus* |  |  | ● |  | MK777751 |
|  |  | *Parascolopsis inermis* |  |  | ● |  | MK777752 |
|  |  | *Pentapodus emeryii* |  |  | ● |  | MK777753 |
|  |  | *Pentapodus setosus* |  | ● | ● |  | MK777754–MK777755 |
|  |  | *Scolopsis affinis* |  |  | ● |  | MK777757 |
|  |  | *Scolopsis bilineata* |  |  | ● |  | MK777758 |
|  |  | *Scolopsis ciliata* |  |  | ● |  | MK777759 |
|  |  | *Scolopsis monogramma* |  | ● | ● |  | MK777760–MK777762 |
|  |  | *Scolopsis taenioptera* |  |  | ● | ● | MK777763–MK777768 |
|  |  | *Scolopsis vosmeri** |  | ● | ● |  | MK777769–MK777771 |
|  |  | *Scolopsis vosmeri*** |  |  |  | ● | MK777772 |
| Syngnathiformes | Callionymidae | *Callionymus curvicornis* |  | ● |  |  | MK777782 |
|  |  | *Callionymus formosanus* | NR |  | ● |  | MK777783–MK777785 |
|  |  | *Callionymus kaianus* |  |  | ● |  | MK777786 |
|  |  | *Callionymus meridionalis* |  |  |  | ● | MK777787 |
|  |  | *Callionymus octostigmatus* | NR |  |  | ● | MK777788 |
|  |  | *Callionymus* sp. 01 | ND |  | ● |  | MK777789 |
|  |  | *Callionymus* sp. 02 | ND |  | ● |  | MK777790 |
|  |  | *Callionymus* sp. 03 | ND |  |  | ● | MK777781 |
|  |  | *Dactylopus dactylopus* |  |  | ● |  | MK777791 |
|  |  | *Eleutherochir opercularis* | NR |  | ● |  | MK777792 |
|  | Centriscidae | *Centriscus scutatus* |  |  | ● |  | MK777793 |
|  | Dactylopteridae | *Dactyloptena orientalis* |  |  | ● |  | MK777794 |
|  |  | *Dactyloptena peterseni* | NR |  | ● |  | MK777795 |
|  | Fistulariidae | *Fistularia petimba** |  |  | ● | ● | MK777796–MK777800 |
|  |  | *Fistularia petimba*** |  |  | ● |  | MK777801 |
|  | Mullidae | *Parupeneus barberinoides* |  |  | ● |  | MK777802 |
|  |  | *Parupeneus forsskali* | NR |  | ● |  | MK777803 |
|  |  | *Parupeneus heptacanthus* |  |  |  | ● | MK777804–MK777805 |
|  |  | *Parupeneus multifasciatus* |  |  | ● |  | MK777806 |
|  |  | *Upeneus guttatus* | NR | ● | ● |  | MK777807–MK777808 |
|  |  | *Upeneus margarethae* | NR |  |  | ● | MK777809–MK777810 |
|  |  | *Upeneus sulphureus* |  |  |  | ● | MK777811–MK777812 |
|  |  | *Upeneus sundaicus* |  | ● |  | ● | MK777813–MK777814 |
|  |  | ***Upeneus tragula*** |  | ● | ● | ● | MK777815–MK777817 |
|  | Syngnathidae | *Hippocampus trimaculatus* |  |  | ● |  | MK777818 |
| Tetraodontiformes | Monacanthidae | *Aluterus monoceros* |  | ● | ● |  | MK777821–MK777822 |
|  |  | ***Monacanthus chinensis*** |  | ● | ● | ● | MK777823–MK777826 |
|  |  | *Paramonacanthus choirocephalus* | NR |  | ● | ● | MK777827–MK777829 |
|  |  | *Paramonacanthus pusillus* |  |  | ● | ● | MK777830–MK777831 |
|  |  | *Thamnaconus hypargyreus* | NR |  | ● |  | MK777832 |
|  | Aracanidae | *Kentrocapros flavofasciatus* | NR |  | ● |  | MK777819 |
|  | Diodontidae | *Cyclichthys orbicularis* |  |  | ● |  | MK777820 |
|  | Ostraciidae | *Lactoria cornuta* |  |  | ● |  | MK777833 |
|  |  | *Tetrosomus gibbosus* |  |  | ● |  | MK777834 |
|  | Tetraodontidae | *Arothron hispidus* |  |  | ● |  | MK777835 |
|  |  | *Chelonodon patoca* |  |  |  | ● | MK777836 |
|  |  | *Lagocephalus cheesemanii* | NR |  | ● |  | MK777837 |
|  |  | ***Lagocephalus spadiceus*** |  | ● | ● | ● | MK777838–MK777842 |
|  |  | *Takifugu poecilonotus* | NR | ● |  |  | MK777843 |
|  |  | *Torquigener hypselogeneion* |  |  | ● |  | MK777844 |
|  | Triodontidae | *Triodon macropterus* |  |  | ● |  | MK777845 |
| Uranoscopiformes | Ammodytidae | *Bleekeria mitsukurii* | NR |  | ● |  | MK777847–MK777848 |
|  | Pinguipedidae | *Kochichthys flavofasciatus* | NR |  | ● |  | MK777849 |
|  |  | *Parapercis alboguttata* |  |  | ● |  | MK777850 |
|  |  | *Parapercis* sp. 01 | ND |  |  | ● | MK777851 |
|  | Uranoscopidae | *Uranoscopus bicinctus* |  | ● | ● |  | MK777852–MK777853 |
|  |  | *Uranoscopus oligolepis* |  | ● | ● |  | MK777854–MK777857 |
| Zeiformes | Zeidae | *Zenopsis nebulosa* |  |  | ● |  | MK777858 |
| 43 | 113 | 478 (458) | 59/  32 | 139 (135) | 285 (274) | 171 (161) |  |

NR–New recoded species in Vietnam; ND–Not yet determined; N, C, S–northern, central, and southern areas in Vietnam; *and** 10 species with intraspecific distances more than 2% (in Table 3); Species in bold were distributed in all areas; ● species recorded in this study; ○ species recorded from Nguyen et al. (2018); numbers in parentheses represent total species excluding those recorded in Nguyen et al. (2018).

Source:

Nguyen ML, Nguyen VQ, Pham VC, Dao HL, Dinh VN, Dam TL. DNA barcoding application mitochondrial COI gene to identify some fish species of family Gobiidae in Vietnam. Journal of Marine Science and Technology. 2018;18(4):443-51.
