# Supplementary material for: DNA barcoding of coastal ray-finned fishes in Vietnam
Source: PLoS One. 2019 Sep 19;14(9):e0222631. doi: 10.1371/journal.pone.0222631 (PMC6752846; doi:10.1371/journal.pone.0222631)

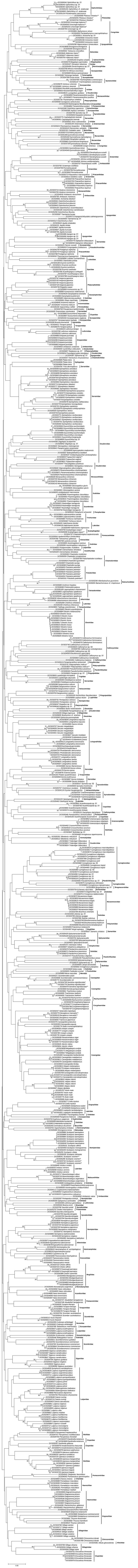

Supplement: S2 Fig — *and** denote 10 species with intraspecific distances more than 2%. (PDF) [file pone.0222631.s002.pdf]
